# Bacterial amylases enable glycogen degradation by the vaginal microbiome

**DOI:** 10.1038/s41564-023-01447-2

**Published:** 2023-08-10

**Authors:** Dominick J. Jenkins, Benjamin M. Woolston, M. Indriati Hood-Pishchany, Paula Pelayo, Alyssa N. Konopaski, M. Quinn Peters, Michael T. France, Jacques Ravel, Caroline M. Mitchell, Seth Rakoff-Nahoum, Christopher Whidbey, Emily P. Balskus

**Affiliations:** 1 Department of Chemistry and Chemical Biology, Harvard University, Cambridge, MA USA; 2 Department of Chemical Engineering, Northeastern University, Boston, MA USA; 3 Division of Infectious Diseases and Division of Gastroenterology, Department of Pediatrics, Boston Children’s Hospital, Boston, MA USA; 4 Department of Microbiology, Harvard Medical School, Boston, MA USA; 5 Department of Chemistry, Seattle University, Seattle, WA USA; 6 Institute for Genome Sciences, University of Maryland School of Medicine, Baltimore, MD USA; 7 Department of Microbiology and Immunology, University of Maryland School of Medicine, Baltimore, MD USA; 8 Vincent Center for Reproductive Biology, Massachusetts General Hospital, Boston, MA USA; 9 Harvard Medical School, Boston, MA USA; 10 Howard Hughes Medical Institute, Harvard University, Cambridge, MA USA

**Keywords:** Hydrolases, Polysaccharides, Bacterial genes, Bacterial pathogenesis, Microbiome

## Abstract

The human vaginal microbiota is frequently dominated by lactobacilli and transition to a more diverse community of anaerobic microbes is associated with health risks. Glycogen released by lysed epithelial cells is believed to be an important nutrient source in the vagina. However, the mechanism by which vaginal bacteria metabolize glycogen is unclear, with evidence implicating both bacterial and human enzymes. Here we biochemically characterize six glycogen-degrading enzymes (GDEs), all of which are pullanases (PulA homologues), from vaginal bacteria that support the growth of amylase-deficient *Lactobacillus crispatus* on glycogen. We reveal variations in their pH tolerance, substrate preferences, breakdown products and susceptibility to inhibition. Analysis of vaginal microbiome datasets shows that these enzymes are expressed in all community state types. Finally, we confirm the presence and activity of bacterial and human GDEs in cervicovaginal fluid. This work establishes that bacterial GDEs can participate in the breakdown of glycogen, providing insight into metabolism that may shape the vaginal microbiota.

## Abstract (web summary)

Biochemical characterization of glycogen-degrading enzymes in vaginal bacteria reveals that the vaginal microbiota possesses the ability to metabolize glycogen in this environment.

## Main

Dysbiosis within the human vaginal microbiota is associated with adverse health outcomes^1^. The bacterial community composition can be classified taxonomically into one of five community state types (CSTs)^2^. CST I–III and V are dominated by a single species of *Lactobacillus*: *L. crispatus, L. gasseri, L. iners* and *L. jensenii*, respectively. By contrast, CST IV consists of a diverse group of anaerobic and facultative anaerobic microbes, including species of *Gardnerella, Prevotella, Mobiluncus* and low levels of *Lactobacillus*. The *Lactobacillus-*dominated CSTs are associated with a vaginal pH below 4.5, low Nugent scores and reduced inflammation^3^, whereas CST IV is associated with a higher pH and several health sequelae, including HIV acquisition^4^, bacterial vaginosis^5^ and preterm birth^6^. However, it is important to note that CST IV is overrepresented in healthy Hispanic and Black women and is not necessarily indicative of dysbiosis^7^. Overall, it has become clear that vaginal microbiota composition alone is insufficient to predict health outcomes and that gaining a mechanistic understanding of this community requires deciphering vaginal bacterial functions^1^.

One function known to influence the composition and stability of host-associated bacterial communities is the liberation of carbohydrates from dietary or host-derived sources by glycoside hydrolases. While this is well established within the human gut microbiota^8–11^, carbohydrate metabolism in the vaginal environment is poorly understood. It is widely believed that glycogen released by exfoliated and lysed epithelial cells supports colonization of vaginal lactobacilli^12,13^ since glycogen levels in vaginal samples are associated with *Lactobacillus* dominance and low vaginal pH^14^. However, until recently, attempts to obtain vaginal *Lactobacillus* isolates capable of growth on glycogen were largely unsuccessful^15,16^, raising the question of whether and how vaginal bacteria access this carbon source.

Glycogen consists of linear chains of *α*-1,4-glycosidic-linked glucose units, with periodic *α*-1,6-glycosidic branches. Metabolism of glycogen requires extracellular glycoside hydrolases to release shorter glucose polymers (maltodextrins). Several vaginal lactobacilli use maltodextrins for growth, leading to an initial hypothesis that a non-*Lactobacillus* glycoside hydrolase in the vaginal environment releases these oligosaccharides^17^. The detection of human α-amylase in cervicovaginal lavage samples (CVLs) may support this proposal^17,18^. But how human amylase, which is produced predominantly in the pancreas and salivary glands^17^, is found in genital fluid has not yet been established.

In addition to human amylase, recent work identified other glycogen-degrading enzymes in vaginal fluid, including glucosidases from the parasite *Trichomonas vaginalis* and several uncharacterized bacterial enzymes detected via proteomics^19–21^. Most notably, a putative secreted Type 1 pullulanase (PulA, EEU28204.2) from *L. crispatus* has been suggested as a candidate glycogen-degrading enzyme (GDE) on the basis of strain-to-strain variation in its predicted signal peptide (SP), which correlates with growth on glycogen^22,23^.

Pullulanases hydrolyse the *α*-1,6-glycosidic bonds in pullulan and other branched oligosaccharides, releasing maltodextrins^24^. Homologues of PulA are encoded in various vaginal bacterial genomes^25^, suggesting that this enzyme is not limited to *L. crispatus* and highlighting the potential for bacterial competition for glycogen. Notably, proteomics studies have identified putative pullulanases from *L. iners* and *Gardnerella vaginalis* in CVLs, but their activity was not biochemically validated^21^. The predicted *α*-1,6-glycosidic bond specificity of pullulanases raises questions regarding the fate of the remaining glycogen backbone and how longer branches are hydrolysed. The identification of these bacterial enzymes also raises questions about the relative role of human amylase in the vaginal ecosystem. Clearly, biochemical characterization of vaginal bacterial enzymes is needed to enhance our understanding of glycogen metabolism in this environment.

Here we report the biochemical characterization of six PulA homologues from vaginal bacteria representing *Lactobacillus-*dominated CSTs (I and III) and the diverse CST IV. Our study reveals that despite a common annotation, these enzymes exhibit variability in their pH profiles, glycogen breakdown product profiles, substrate preferences and susceptibility to inhibitors. By analysing multi-omics datasets, we reveal that the genes encoding these GDEs are present and transcribed in all CSTs. Using activity-based protein profiling (ABPP)^26^ and a selective enzymatic assay, we demonstrate that both human and bacterial GDEs are present and active in cervical vaginal fluid (CVF). Overall, this work provides molecular insight into the bacterial metabolism of an abundant carbon source in the vaginal microbiota.

## Results

### Bioinformatic identification and analysis of bacterial extracellular GDEs

To identify candidate vaginal bacterial GDEs, we conducted a BLASTp search of 151 vaginal isolate genomes in the IMG database using the *L. crispatus* PulA (EEU28204.2) as a query sequence^22^, with a cut-off of 35% amino acid identity. Hits were further narrowed to those containing both a glycoside hydrolase domain and a signal peptide, since glycogen degradation occurs extracellularly^27^. A total of 62 homologues were identified in strains from 11 bacterial species (Supplementary File 1), including *L. crispatus* (7/9 strains in the database, average 99% amino acid identity to our query), *L. iners* (12/13, 45%), *Mobiluncus mulieris* (2/4, 43%), *Prevotella bivia* (2/2, 40%) and *G. vaginalis* (15/18, 37%). Gene neighbourhood analysis revealed another signal peptide-containing glycoside hydrolase (GH13) encoded next to the *P. bivia pulA* (25% identity to PulA), so this sequence was also included. Subsequent characterization efforts focused on this set of proteins. We also detected potential homologues with lower identity (Supplementary File 1), including one from *Streptococcus agalacticae* and one significantly smaller protein in *G. vaginalis* homologous to a recently reported α-glucosidase enzyme that is active on maltose and other oligosaccharides but does not degrade glycogen^28^.

**Fig. 1:**
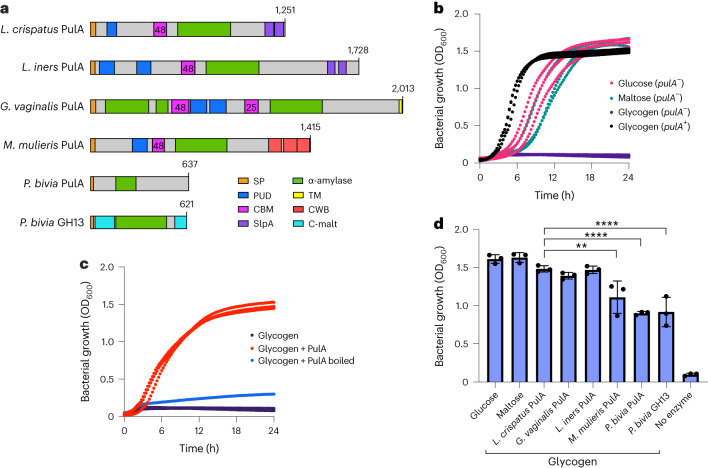
Purified bacterial GDEs support *L. crispatus* growth on glycogen. **a**, Predicted domains in putative vaginal bacterial extracellular GDEs. α-amylase, α-amylase catalytic domain (GH13); C-malt, cyclomaltodextrinase domain. **b**, Growth of *L. crispatus* C0176A1 (*pulA*^−^, JAEDCG000000000) and MV-1A-US (*pulA*^+^) on different carbon sources. **c**, *L. crispatus* C0176A1 (*pulA*^−^) grown on oyster glycogen supplemented with 200 nM purified *L. crispatus* PulA. **d**, 24 h OD_600_ values of *L. crispatus* C0176A1 (*pulA*^−^) grown on glucose, maltose or glycogen supplemented with 200 to 400 nM purified protein (*L. crispatus* PulA vs *M. mulieris* PulA, *P* = 0.0084; *L. crispatus* PulA vs *P. bivia* PulA, *P* < 0.0001; *L. crispatus* PulA vs *P. bivia* GH13, *P* < 0.0001). All growth curves are representative of three experimental replicates (*n* = 3). Error bars represent one standard deviation above and below the mean of all data collected. A multiple comparisons (Tukey) one-way analysis of variance (ANOVA) was performed to determine statistical significance. **P ≤ 0.01, ****P ≤ 0.0001. Source data

PFAM domain analysis revealed that all six candidate GDEs contain an S-layer protein A domain (SlpA), a cell-wall binding domain (CWB) or transmembrane helices (TM), suggesting localization on the cell surface^29–31^. In addition, each protein contains at least one α-amylase catalytic domain (PF00128), a member of the glycoside hydrolase 13 enzyme family known to cleave various glycosidic bonds^32^. Interestingly, the *G. vaginalis* enzyme has two amylase domains. Several enzymes possess putative carbohydrate-binding domains common to bacterial enzymes in this class, including the pullulanase domain (PUD; PF03714)^33^. Additional carbohydrate-binding modules (CBMs) from the CAZy database found in these enzymes include CBM25 and CBM48, which are involved in binding different linear and cyclic α-glucans related to starch and glycogen^32,34^ and in multivalent binding to soluble amylopectin and pullulan^35^ (Fig. 1a).

### Purified GDEs support the growth of *L. crispatus* on glycogen

**Extended Data Fig. 1:**
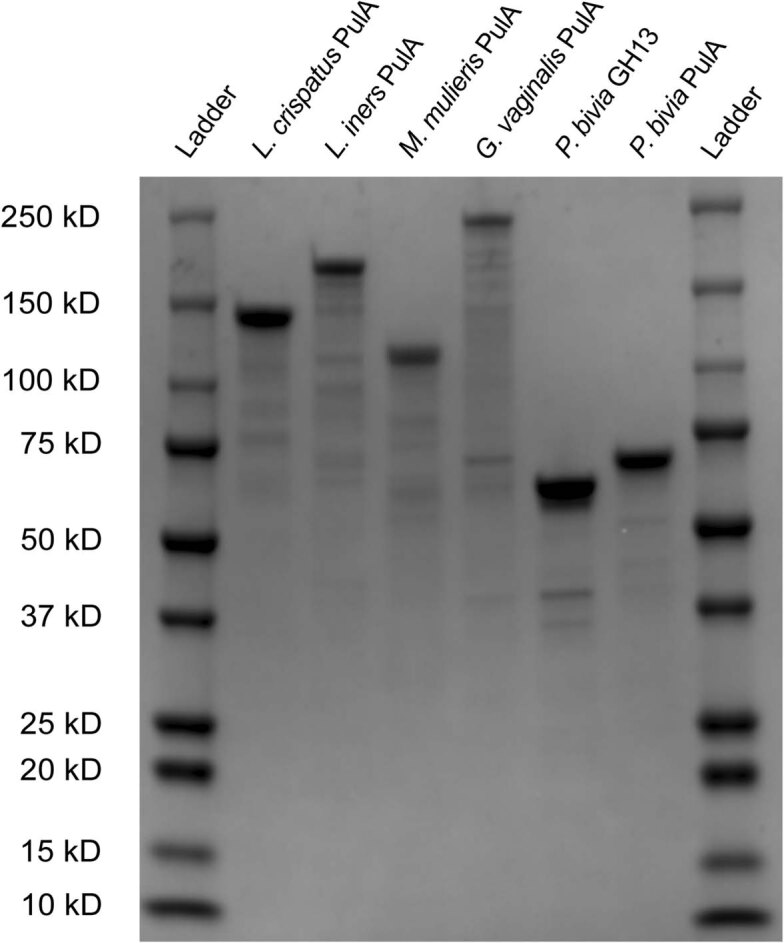
SDS page analysis of purified extracellular GDEs. 2 μg of purified protein was loaded onto a Biorad (2-14% Bis-Tris) SDS page gel. From left to right, Precision Plus Protein All Blue Standards (BioRad); *L. crispatus* PulA, 137kD; *L. iners* PulA, 192kD; *M. mulieris* PulA, 151kD; *G. vaginalis* PulA, 219kD; *P. bivia* GH 13, 70.4kD; *P. bivia* PulA, 72.8kD; Precision Plus Protein All Blue Standards (BioRad). Molecular weights of heterologously expressed proteins were predicted in EXPASY. This experiment was repeated twice demonstrating similar results (n = 2). Source data

**Extended Data Fig. 2:**
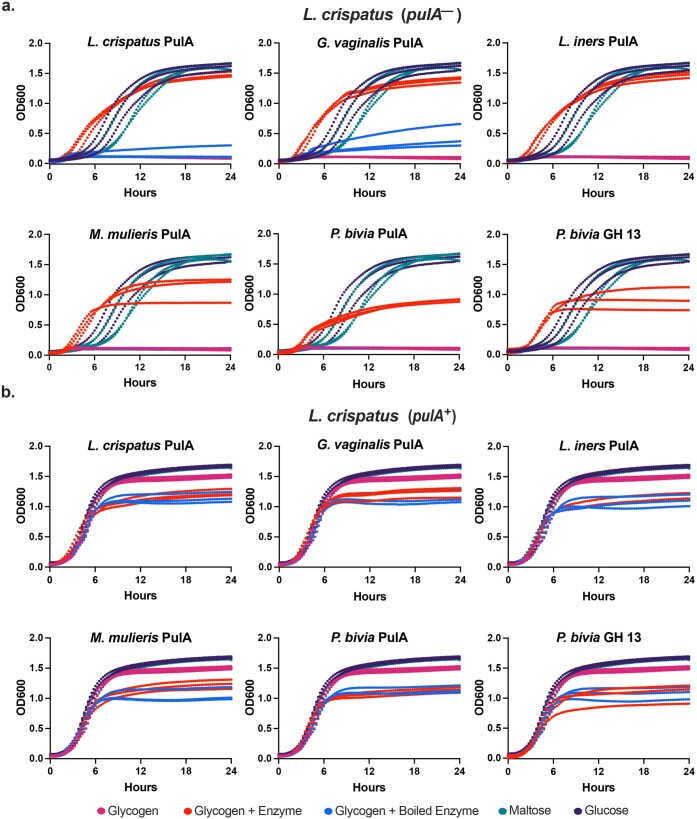
Recovery of *L. crispatus* growth on glycogen with putative PulA homolog addition. **a**. Growth of *L. crispatus* C0176A1 (*pulA*^*—*^) containing all relevant controls for protein complementation assays b. Growth of *L. crispatus* MV-1A-US (*pulA*^*+*^) containing all relevant controls for protein complementation assays. Glycogen, maltose, and glucose conditions are derived from the same data across all graphs within each panel. All growth data consists of three independent experiments performed over three days (n = 3). Source data

To examine their ability to degrade glycogen, we heterologously expressed and purified each enzyme (Extended Data Fig. 1 and Supplementary Fig. 1), then tested whether it rescued growth of a *pulA*-deficient *L. crispatus* strain on glycogen (Fig. 1b,c and Extended Data Fig. 2). Addition of purified *L. crispatus* PulA to the medium restored growth, providing direct evidence that PulA is sufficient for *L. crispatus* glycogen metabolism^22^ (Fig. 1c and Extended Data Fig. 2). The other enzymes also supported growth (Fig. 1d and Extended Data Fig. 2), although the lower densities of cultures grown with the *M. mulieris* PulA and *P. bivia* enzymes suggest that they are not as efficient at glycogen degradation, or that their specific oligosaccharide products are not as accessible to *L. crispatus*.

### Purified GDEs have unique substrate preferences

**Extended Data Fig. 3:**
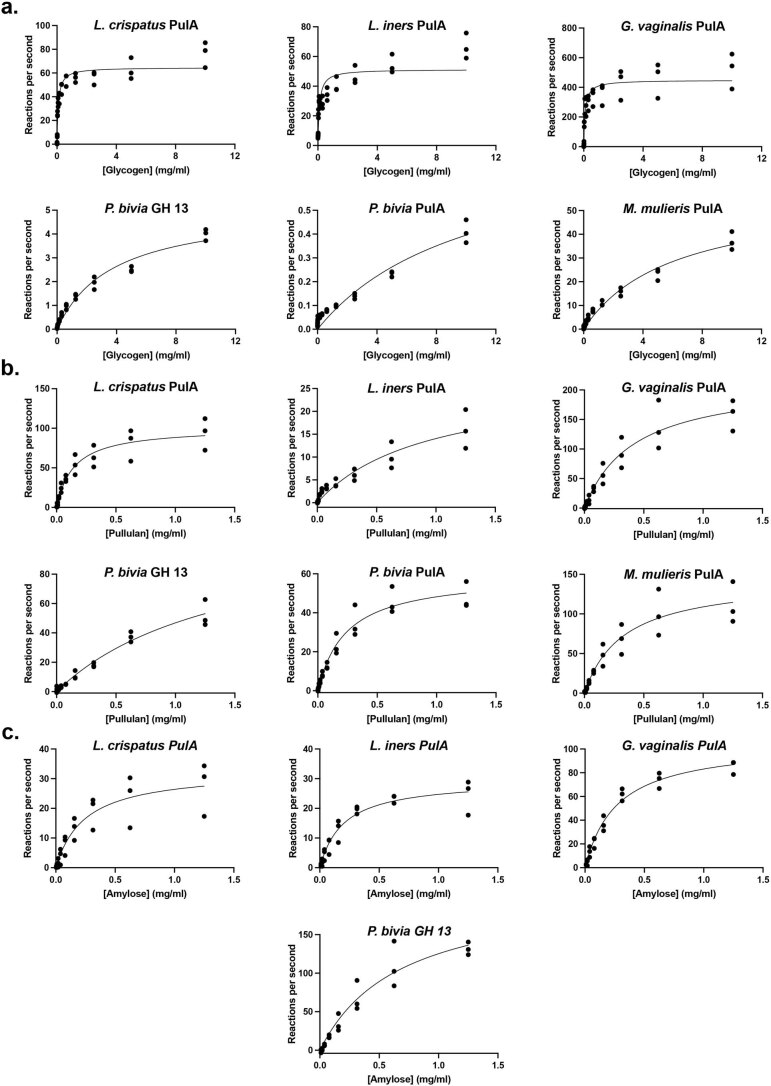
Kinetic analysis of vaginal bacterial GDEs. **a**. Michaelis–Menten kinetic analysis for assays with glycogen. **b**. Michaelis–Menten kinetic analysis for assays with pullulan **c**. Michaelis–Menten kinetic analysis for assays with amylose. All data are representative of three experimental replicates performed over two days. Nonlinear fitting was performed in Graphpad Prism 8. Source data

**Extended Data Fig. 4:**
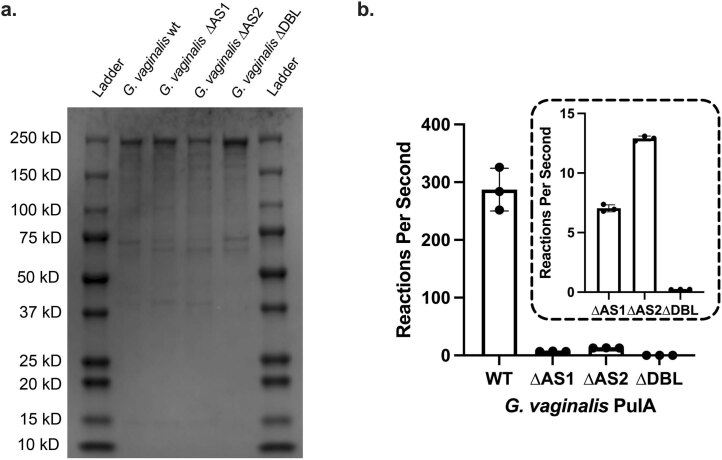
Active site mutants of *G. vaginalis* PulA. **a**. 2 μg of active site mutants and wild type *G. vaginalis* PulA was loaded onto a Biorad (2-14% Bis-Tris) SDS page gel. This experiment was repeated twice demonstrating similar results (n = 2). **b**. Specific activity of active site mutants on glycogen. Inset graph shows specific activity data for *G. vaginalis* mutants. ∆AS1, ∆AS2, and ∆DBL represents D233A, D1317A, and both mutations respectively. Data is representative of three experimental replicates over two days. Error bars represent one standard deviation above and below the mean. Source data

We next measured the kinetics of breakdown of a variety of glucose polymers to determine each enzyme’s substrate preference and specificity for different glycosidic linkages (Table 1 and Extended Data Fig. 3). In addition to glycogen, we tested amylose, which consists solely of *α*-1,4-glycosidic bonds and pullulan, which consists of maltotriose units connected by *α*-1,6-glycosidic bonds. All enzymes were active on glycogen (Table 1 and Extended Data Fig. 3). Interestingly, mutants of the *G. vaginalis* enzyme with either of the two amylase domains inactivated retained only 5% of wild-type activity, suggesting that the two domains may act synergistically (Extended Data Fig. 4). All enzymes were active on pullulan, suggesting that they cleave *α*-1,6-glycosidic bonds but differed in their activity towards the *α*-1,4-glycosidic bonds in amylose, with *L. crispatus, L. iners*, *G. vaginalis* and *P. bivia* GH13 enzymes showing activity, while *M. mulieris* and *P. bivia* PulA enzymes were inactive (Table 1 and Extended Data Fig. 3).

**Table 1:** Kinetic analysis of vaginal bacterial glycogen-degrading enzymes on various carbohydrate polymers at pH 5.5

Enzyme	Substrate	*k*_cat_ (s^−1^)	*K*_m_ (mg ml^−1^)	Specificity constant (ml mg^−1^ s^−1^)	Classification
*L. crispatus* PulA	Glycogen	65 ± 4	0.091 ± 0.026	710 ± 210	Type II pullulanase (Amylopullulanase)
	Amylose	33 ± 7	0.25 ± 0.14	130 ± 80	
	Pullulan	100 ± 10	0.15 ± 0.05	700 ± 250	
*L. iners* PulA	Glycogen	51 ± 5	0.10 ± 0.05	500 ± 220	Type II pullulanase (Amylopullulanase)
	Amylose	30 ± 4	0.20 ± 0.07	150 ± 60	
	Pullulan	27 ± 9	0.93 ± 0.56	29 ± 20	
*G. vaginalis* PulA	Glycogen	450 ± 40	0.098 ± 0.044	4,600 ± 2,100	Type II pullulanase (Amylopullulanase)
	Amylose	110 ± 10	0.27 ± 0.06	390 ± 90	
	Pullulan	220 ± 40	0.42 ± 0.170	520 ± 230	
*M. mulieris* PulA	Glycogen	57 ± 9	6.1 ± 1.7	9.5 ± 3.0	Type I pullulanase
	Amylose	NA	NA	NA	
	Pullulan	150 ± 20	0.33 ± 0.14	440 ± 200	
*P. bivia* PulA	Glycogen	0.81 ± 0.31	11 ± 7	0.077 ± 0.058	Type I pullulanase
	Amylose	NA	NA	NA	
	Pullulan	60 ± 6	0.24 ± 0.07	250 ± 70	
*P. bivia* GH13	Glycogen	5.1 ± 0.60	3.8 ± 1.0	1.4 ± 0.3	Pullulan hydrolase type I (Neopullulanase)
	Amylose	210 ± 50	0.68 ± 0.33	310 ± 160	
	Pullulan	120 ± 30	1.5 ± 0.6	79 ± 38	

Values are representative of three independent experiments over 2 d (*n* = 3). NA, not applicable due to lack of activity. Error range represents one standard deviation.

Source data

The measured kinetic parameters of these GDEs were broadly consistent with those of other bacterial enzymes that process these substrates (glycogen^36–38^, amylose^39,40^, pullulan^39,41^). Comparing the specificity constants (*k*_cat_/*K*_m_) for each substrate revealed that glycogen is the preferred substrate for the *G. vaginalis* and *L. iners* enzymes. The *L. crispatus* PulA had similar specificity constants for both pullulan and glycogen, with activity towards amylose. *P. bivia* PulA and *M. mulieris* PulA had higher specificity constants for pullulan compared with glycogen and amylose, while the *P. bivia* GH13 enzyme preferred amylose (Table 1 and Extended Data Fig. 3). Overall, these enzymes varied in their substrate preference despite sharing homology with *L. crispatus* PulA.

### *Lactobacillus* GDEs maintain activity at low pH

**Fig. 2:**
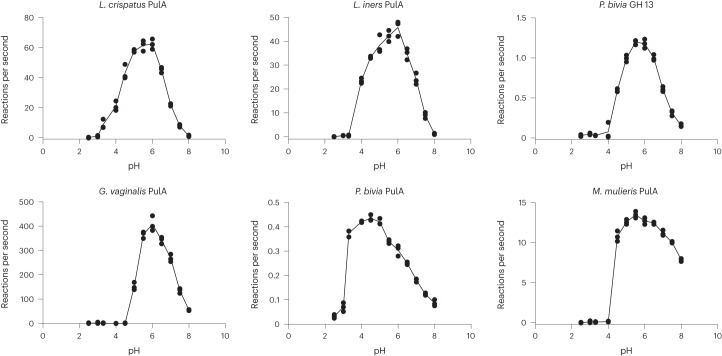
*Lactobacillus* amylopullulanases are adapted to a low pH environment. pH profiles of six extracellular glycogen-degrading enzymes. Buffer systems consisted of glycine (pH = 2.5–3.3), sodium acetate (pH = 4.0–5.5), MES (pH = 6.0–6.5) and HEPES (pH = 7.0–8.0). Data are representative of three independent experiments over 2 d (*n* = 3). Source data

*Lactobacillus*-dominant CSTs are typically associated with a low vaginal pH (<4.5)^42^ due to production of lactic acid^14^. We therefore hypothesized that GDEs from lactobacilli may have evolved to maintain activity at a lower pH than those from other vaginal bacteria. We measured GDE activity on glycogen over a pH range of 2.5–8.0 (Fig. 2). Five of the GDEs exhibited maximum activity between pH 5.5 and 6.0, which is consistent with other characterized bacterial pullulanases and amylopullulanases^43^. *P. bivia* PulA exhibited maximum activity at a slightly lower pH between 4.5 and 5 (Fig. 2). Most of the enzymes from vaginal anaerobes—*G. vaginalis* PulA, *M. mulieris* PulA and *P. bivia* GH13—showed almost no activity at pH 4.0. Critically, however, the *L. crispatus*, *L. iners* and *P. bivia* PulAs displayed 34%, 51% and 97% of their maximal activity at pH 4.0, suggesting that they are better adapted to a low pH environment. This activity may explain how vaginal lactobacilli can utilize host-derived glycogen under low pH conditions, potentially contributing to their dominance.

### GDEs produce distinct oligosaccharide profiles

**Fig. 3:**
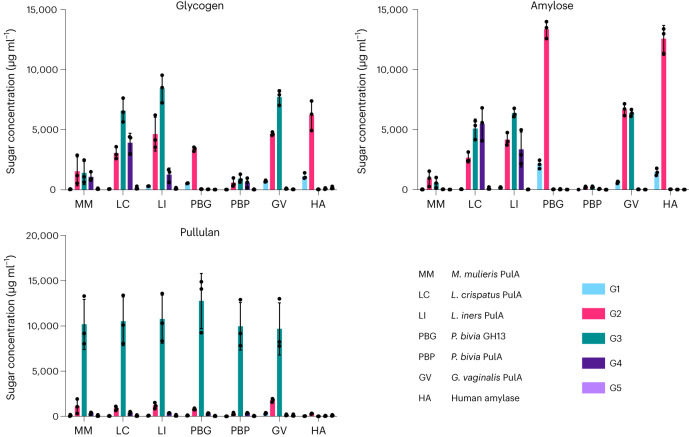
GDEs from different vaginal lactobacilli produce unique breakdown product profiles. Polymer breakdown products generated following overnight incubation with purified enzyme. LC–MS analysis is representative of three independent experiments performed over 3 d (*n* = 3). Error bars represent one standard deviation above and below the mean. G1, glucose; G2, maltose and isomers; G3, maltotriose and isomers; G4, maltotetraose and isomers; G5, maltopentaose and isomers. Source data

We next sought to characterize and quantify the specific oligosaccharide products of each enzyme (Fig. 3). Both human amylase and the *P. bivia* GH13 produced predominantly glucose disaccharides (G2) and a small amount of glucose (G1) from glycogen and amylose. In contrast, the enzymes annotated as Type I Pullulanases produced longer oligosaccharides in addition to G2, including glucose trisaccharides (G3) and in some cases glucose tetrasaccharides (G4). These results resemble those observed for previously characterized bacterial amylopullulanses^44,45^. G4 was not detected in assays with *G. vaginalis* PulA and was only detected at a low level in assays with the *M. mulieris* and *P. bivia* enzymes. However, the *Lactobacillus*-derived PulA enzymes produced a higher relative amount of G4 when acting on amylose or glycogen. During incubation with pullulan, all bacterial enzymes produced predominantly G3, whereas the human salivary amylase was inactive. The sole production of G3 is common among pullulan-degrading enzymes^41,43,44,46^. Notably, pullulanase activity appears unique to vaginal bacterial GDEs and is not exhibited by the human enzyme (Table 1 and Fig. 3).

**Extended Data Fig. 5:**
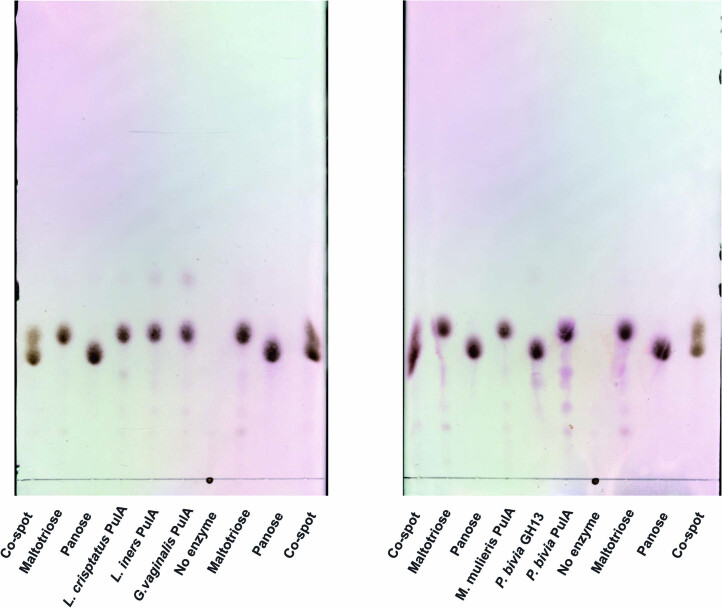
Enzymatic digestion of pullulan by vaginal bacterial glycogen-degrading enzymes and resolution of maltotriose isomers by thin layer chromatography (TLC). 1 μL of each standard (10 mg/mL) and enzymatic reaction mixture was spotted onto a TLC plate (20 cm by 20 cm, Analtech Silica gel HLF). TLC was run for approximately 5 h in 3:2:1 butanol: acetic acid: water and stained with 1:19 sulfuric acid: ethanol. These results are representative of two trials showing similar findings (n = 2).

The specific G3 products of pullulan degradation were identified using thin-layer chromatography (TLC). Every enzyme except *P. bivia* GH13 produced maltotriose, confirming their ability to cleave α-1,6-glycosidic bonds. *P. bivia* GH13 produced panose, suggesting that this enzyme cleaves only *α*-1,4-glycosidic bonds in pullulan (Extended Data Fig. 5). These data, paired with the kinetic analyses (Table 1), demonstrate that both *Lactobacillus* PulA enzymes and the *G. vaginalis* PulA enzyme can cleave the *α*-1,4 and *α*-1,6-glycosidic bonds found in glycogen and support their reassignment as type II pullulanases or amylopullulanases (EC. 3.2.1.1/41, reviewed in ref. ^45^). *P. bivia* GH13 only cleaves *α*-1,4-glycosidic linkages (including within pullulan), identifying this enzyme as a pullulan hydrolase type I or neopullulanase (EC 3.2.1.135, reviewed in ref. ^47^). In contrast, the lack of activity of the *M. mulieris* and *P. bivia* PulA enzymes towards amylose identifies them as type I pullulanases (EC 3.2.1.41) and may explain their reduced ability to complement *L. crispatus* growth on glycogen (Figs. 1d and 3, Table 1 and Extended Data Fig. 5).

### Acarbose selectively inhibits GDEs from CST IV bacteria

**Extended Data Fig. 6:**
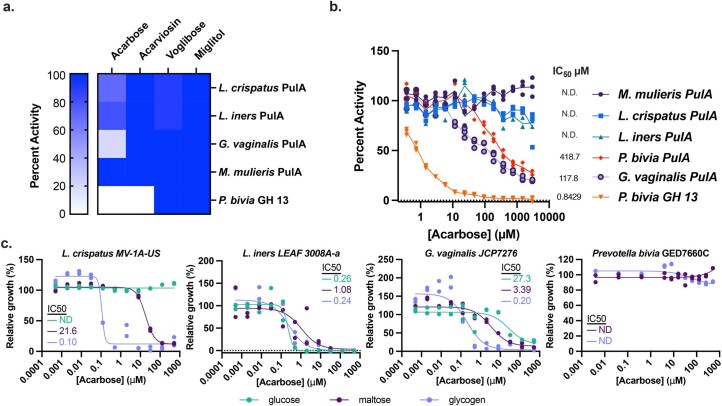
Selective inhibiton of vaginal microbial GDEs. **a**. Effects of known amylase inhibitors (1 mM) on the activities of purified GDEs toward a BODIPY fluorescent starch substrate (n = 1). **b**. Inhibitory activity of acarbose toward purified extracellular amylases. A BODIPY fluorescent starch substrate was used and activity was normalized to a no inhibitor control. Data are representative of three experimental replicates over two days. **c**. Bacteria were grown in the presence of the indicated concentrations of acarbose in media containing either glucose, maltose or glycogen as the primary carbohydrate source. Growth in the presence of inhibitor was normalized to the untreated control. IC_50_ values were calculated using a least-squares regression of the normalized values. ND (Not determined) is indicated when the resulting curve fit was poor and an IC_50_ value could not be confidently determined, or the overall growth inhibition was less than 10%. Data are representative of at least two biological replicates performed over two days. Source data

Given their role in enabling growth on glycogen and the biochemical distinctions between different GDEs, we hypothesized that these enzymes may be targets for possible therapeutic intervention aimed at establishing a *Lactobacillus-*dominant community. Of four clinically used amylase inhibitors, only acarbose and acarviosin showed any activity towards the GDEs (Extended Data Fig. 6a). Acarbose inhibited *G. vaginalis* PulA, *P. bivia* PulA and *P. bivia* GH13 enzymes, with half-maximum inhibitory concentration values (IC_50_) of 120 ± 30 μΜ, 420 ± 90 μΜ and 0.84 ± 0.05 μΜ, respectively, while the *L. crispatus, L. iners* and *M. mulieris* enzymes were largely unaffected (Extended Data Fig. 6b).

Since acarbose selectively inhibited GDEs from CST IV-associated microbes, we characterized its effect on bacterial growth as a first step towards testing its utility for community modulation. While acarbose inhibited *G. vaginalis* growth on glycogen (IC_50_ = 0.2 μM), it also inhibited *L. crispatus* growth on maltose (IC_50_ = 22 μM) and glycogen (IC_50_ = 0.1 μM) even though *L. crispatus* PulA was not affected in vitro. Interestingly, *L. crispatus* growth was not affected when glucose was the primary carbon source (Extended Data Fig. 6c). These data suggest that acarbose inhibits additional *L. crispatus* enzymes involved in maltodextrin metabolism. Despite potently inhibiting the *P. bivia* GH13 in vitro, acarbose had no impact on *P. bivia* growth on any substrate (Extended Data Fig. 6c). This suggests that *P. bivia* PulA, which was less susceptible to inhibition in vitro, is likely the predominant GDE in this organism and that intracellular maltodextrin catabolism in *P. bivia* is not affected by acarbose. Overall, although acarbose is not a suitable candidate for community modulation due to its broad target spectrum, these results highlight differences between the GDEs that may potentially be targeted for selective inhibition.

### GDEs are present in human vaginal sequencing datasets

Having identified bona fide vaginal bacterial GDEs, we next sought to understand the presence and expression of genes encoding these enzymes in the vaginal environment. While other searches have detected putative bacterial amylases in clinical samples using proteomics^21^, metagenomics and metatranscriptomics^23,48^, the activities of these enzymes were not biochemically verified. We employed shortBRED (Short, Better Representative Extract Dataset) to identify biochemically characterized GDEs in a dataset of 178 paired vaginal metagenomes and metatranscriptomes from 40 non-pregnant, reproductive-age women who self-collected vaginal swabs over 10 weeks^48,49^. ShortBRED is a computational tool that identifies and quantifies unique amino acid sequences that are distinct to a query protein (85% identity cut-off). In contrast to previous studies, all enzymes queried are predicted to be extracellular and degrade glycogen in vitro, increasing confidence that any hits also possess this activity.

**Fig. 4:**
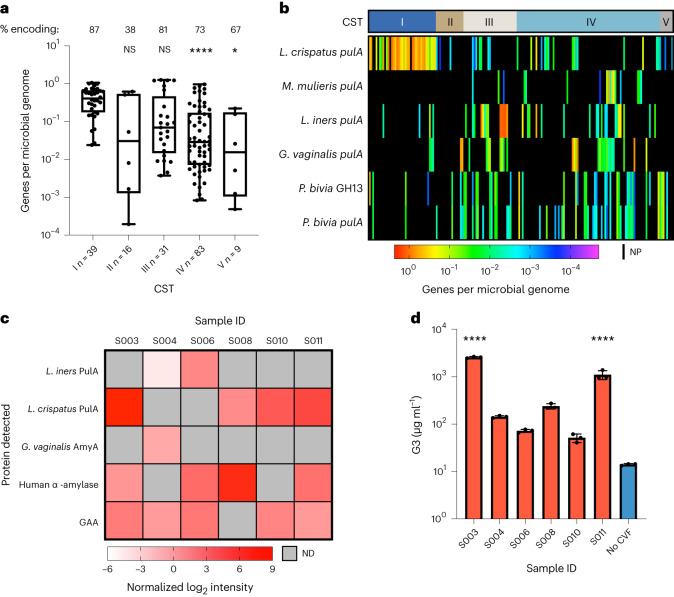
Human CVF samples contain active human amylase and bacterial GDEs. **a**, Metagenomic analysis of 178 participant samples using ShortBRED analysis of biochemically characterized GDEs stratified by CST. Only samples encoding a bacterial GDE were plotted. % encoding represents the percentage of samples that contain >0 genes per bacterial genome. A multiple comparisons (Dunnett) one-way ANOVA was performed to determine statistically significant differences compared to CST I abundance (CST IV, ****P < 0.0001; CST V, *P = 0.0196; ^NS^*P* > 0.05,) The box represents 1.5× the interquartile range and the whiskers represent the minimum to the maximum of the dataset. The centerline denotes the median. **b**, Heat map of metagenomic presence and abundance detected using ShortBRED within each sample. NP, not present. **c**, ABPP analysis identifies bacterial GDEs and human proteins (α-amylase and GAA) in CVF supernatants. ND, not detected; GAA, lysosomal α-glucosidase. **d**, Human CVF contains distinctly bacterial pullulanase activity at pH 5.5. Data are representative of three experimental replicates over 2 d and the error bars are one standard deviation above and below the mean. A multiple comparisons (Dunnett) one-way ANOVA was performed to determine statistically significant differences compared to the no-CVF sample (blue) (S003, ****P < 0.0001; S011, ****P < 0.0001). Source data

**Extended Data Fig. 7:**
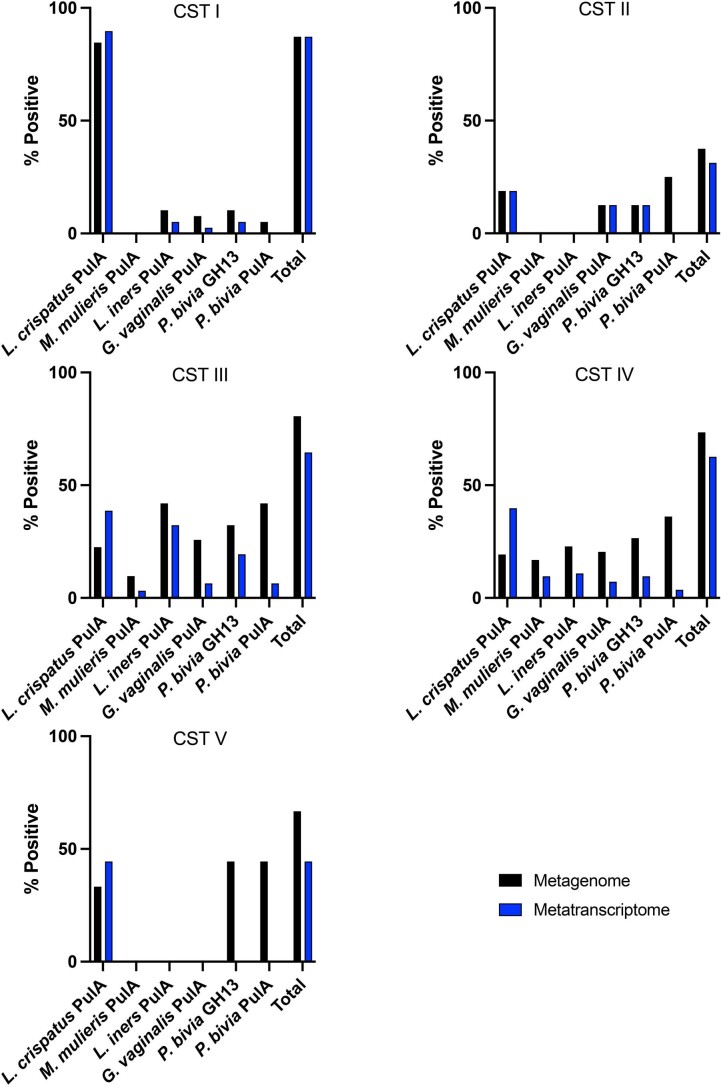
Bacterial GDE metatranscriptomic and metagenomic presence. **% Positive** represents the percentage of samples that contained reads mapping to our query proteins (reads > 0). The sample size is as follows: **CST I**, n = 39; **CST II**, n = 16; **CST III**, n = 31; **CST IV**, n = 83; **CST V**, n = 9. Source data

Combined reads from the six GDEs were more abundant in CST I metagenomes compared with CST II and CST IV metagenomes (Fig. 4a). This increased abundance in CST I samples is due to *L. crispatus pulA* (Fig. 4b), which was detected in 84.6% of the metagenomes and 89.7% of the metatrascriptomes from CST I participants. *M. mulieris pulA* was not detected in these individuals and the other four GDEs were detected in fewer than 11% of metagenomes and metatranscriptomes (Extended Data Fig. 7). In CST III samples, which were dominated by *L. iners*, *L. iners pulA* was detected in 41.9% and 32.3% of the metagenomes and metatranscriptomes, respectively. Interestingly, genes encoding other GDEs (*L. crispatus* PulA, *G. vaginalis* PulA, both *P. bivia* GDEs) were detected in >20% of CST III metagenomes. However, the detection of these genes in the metatranscriptomes was highly variable (6.45%–38.7%).

All GDEs were detected in CST IV metagenomes at frequencies of 16.9%–36.1%. While *L. crispatus pulA* was detected in 39.8% of CST IV metatranscriptomes, transcripts from other GDEs were detected in only 3.6%–10.8% of the datasets (Extended Data Fig. 7). Overall, this analysis demonstrates that characterized bacterial GDEs are present in vaginal metagenomes and expressed in various vaginal bacterial CSTs, with CST III and CST IV communities in particular harbouring GDEs from multiple species.

### Clinical samples contain active human and bacterial enzymes

**Extended Data Fig. 8:**
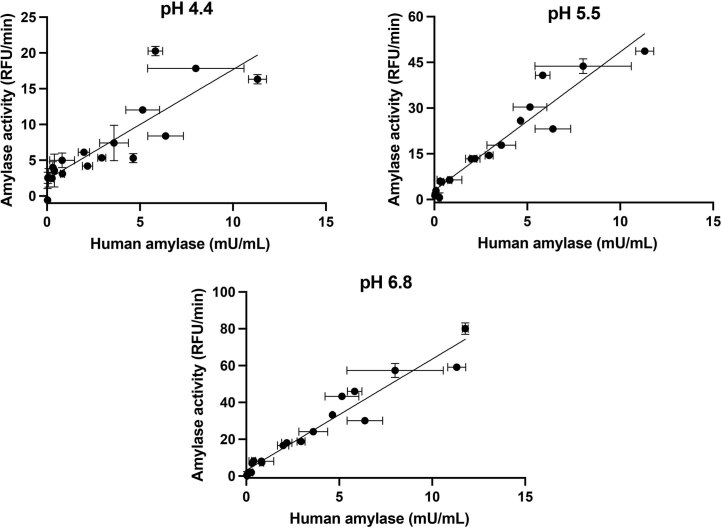
Activity of human CVL supernatants correlates with human amylase levels detected by ELISA. Outliers were determined and removed using a ROUT test. A two-tailed Pearson test was used to determine correlation (pH 4.4, r = 0.8556, 95% CI 0.6472 to 0.9450, p <0.0001; pH 5.5, r = 0.9611, 95% CI 0.8966 to 0.9857, p <0.0001; pH 6.8, r = 0.9713, 95% CI 0.9252 to 0.9891, p <0.0001) Activity data represents the mean of three experiments over two days and the ELISA detection is the mean of two experiments over two days. Error bars represent one standard deviation above and below the mean. Source data

We next sought to detect the activity of bacterial GDEs and human amylase in clinical samples. We initially analysed 20 CVL sample supernatants spanning a range of Nugent scores (0–8), comparing total amylase activity to the concentration of human amylase determined by enzyme-linked immunosorbent assay (ELISA) (Supplementary Fig. 2 and Extended Data Fig. 8). Activity assays with a fluorescent starch substrate were conducted across a range of pHs, spanning the healthy vaginal environment (4.4) to the optimum for the human amylase (6.8). At all pH values, there was a statistically significant correlation between these measurements (Extended Data Fig. 8), suggesting that the majority of the amylase activity is human. However, it is interesting to note that as pH decreased, the correlation coefficient was reduced, perhaps suggesting increased contribution from other enzymes at lower pH (Extended Data Fig. 8). Comparing these results to Nugent scores (low Nugent 0–3, high Nugent 7–10), we found no difference in amylase activity or human amylase levels (Supplementary Fig. 3). To confirm the specificity of the ELISA for the human enzyme, we assayed our purified bacterial enzymes using the same kit and found no cross-reactivity at enzyme concentrations as high as 1 μM (Supplementary Fig. 2). Together, these results indicate that the contribution of human amylase to glycogen degradation should not be overlooked, despite the existence of bacterial enzymes with related activities.

**Extended Data Fig. 9:**
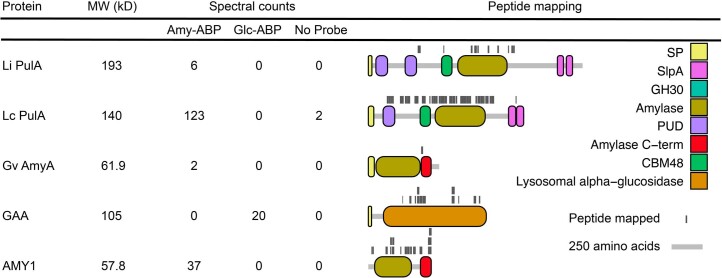
Peptide mapping to proteins identified through ABPP. Spectral counts indicate the sum of total spectra assignable to a protein across all six individual samples. Abbreviations: **Li PulA**, *L. iners* PulA; **Lc PulA**, *L. crispatus* PulA; **Gv AmyA**, *G. vaginalis* AmyA; **GAA**, human lysosomal α-glucosidase; **AMY1**, human salivary amylase; **SP**, signal peptide; **SlpA**, surface layer protein A; **GH**, glycoside hydrolase domain **PUD**, bacterial pullulanase-associated domain; **CBM**, Carbohydrate binding module.

We next attempted to determine whether bacterial GDEs were active in CVF samples by applying activity-based protein profiling (ABPP)^50^ using probes targeting amylase (Amy-ABP) and glucosidase (Glc-ABP) enzymes. After confirming that the purified bacterial GDEs reacted with at least one probe (Supplementary Figs. 4 and 5), CVF supernatants were labelled with biotin-tagged probes, followed by pull-down, tryptic digest and liquid chromatography tandem mass spectrometry (LC–MS/MS) identification of the peptides. The Amy-ABP probe identified *L. crispatus* PulA, *L. iners* PulA and *G. vaginalis* AmyA in these samples (Fig. 4c and Extended Data Fig. 9). *L. crispatus* PulA and *L. iners* PulA were mutually exclusive, consistent with previous studies finding their co-occurrence uncommon^7^. In one sample (S004), we observed co-occurrence of two bacterial enzymes: *L. iners* PulA and *G. vaginalis* AmyA. Although we did not characterize *G. vaginalis* AmyA in this study (because of its low amino acid similarity to *L. crispatus* PulA), it was recently shown to degrade glycogen^51^. We also detected human α-amylase (AMY1) in the majority of samples (Fig. 4c and Extended Data Fig. 9). In contrast to the Amy-ABP-enriched proteins, all Glc-ABP-enriched proteins were human in origin. The protein with the highest intensity was lysosomal α-glucosidase, which is canonically localized to the lysosome but has been detected previously in CVF (Fig. 4c, Extended Data Fig. 9 and Supplementary File 1)^52–54^. Overall, these data demonstrate that bacterial GDEs are present and active in the vaginal environment, including *L. crispatus* PulA, *L. iners* PulA and *G. vaginalis* AmyA.

**Extended Data Fig. 10:**
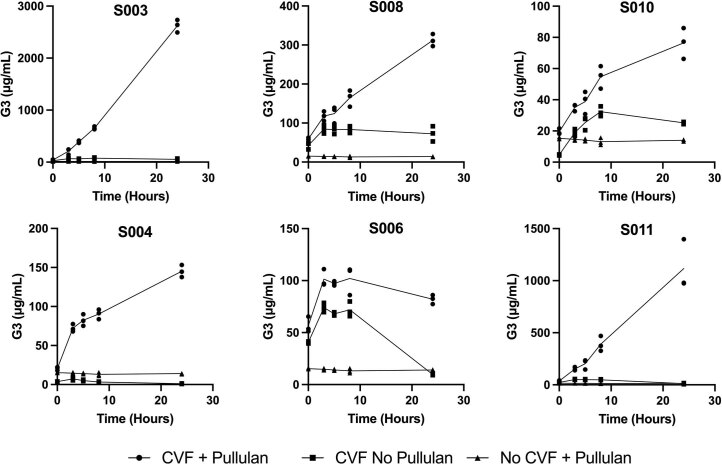
Time-course analysis of CVF sample pullulan degradation confirms enzymatic activity. Timepoints from CVF reactions were removed and frozen at 3, 5, 8, and 24 hours. These data are derived from three experimental replicates over two days. Source data

To further validate the activity of bacterial GDEs in these samples, we used an LC–MS-based assay to detect pullulan degradation, leveraging the observation that all characterized GDEs metabolize pullulan, whereas the human amylase does not (Fig. 3). Every CVF sample showed activity in this assay (Extended Data Fig. 10). Notably, the two most active samples, S003 and S011, had the highest intensity of *L. crispatus* PulA in the ABPP experiment (Fig. 4c) and generated significantly increased levels of G3 compared with a no-CVF control (Fig. 4d). These results further demonstrate that vaginal bacterial GDEs are active in clinical samples and validate a simple, accessible assay for bacterial GDEs that does not depend on proteomic workflows.

## Discussion

In this study, we biochemically characterized six GDEs from vaginal bacteria. Our results demonstrate that in addition to relying on human amylase, some vaginal bacteria possess alternative enzymes for accessing glycogen. These findings are further validated by a separate report of the glycogen-degrading activity of *L. crispatus* PulA (GlgU, 99% identity)^55^. Critically, despite sharing a common annotation, we find that the substrate preferences and breakdown products of bacterial GDEs are quite distinct. This is consistent with the unique carbohydrate-binding modules found in each protein and may suggest adaptation to process structurally distinct glucose polymers in the vaginal environment. Further, since the oligosaccharides produced from glycogen breakdown are released extracellularly and may act as ‘public goods’^56^, the differences in the product distributions of these enzymes may suggest that differential availability of glycogen-derived oligosaccharides between CSTs supports the growth of distinct non-glycogen-degrading bacteria via cross-feeding^11^. A better understanding of the structure of glycogen within the vaginal environment and whether it differs among CSTs is needed to further evaluate this possibility.

Our work also suggests a potential mechanism supporting *L. crispatus* growth and dominance. Specifically, we discovered that *L. crispatus* PulA is active at the low pH values (~3.5–4) associated with vaginal health. This enzymatic activity may therefore enable *L. crispatus* to access glycogen under conditions where the human amylase is minimally active and the growth of competing bacteria is inhibited. Critically, the pH profiles, substrate preferences and breakdown products of amylases cannot be predicted from primary sequence analysis, further highlighting the need for biochemical characterization to support bioinformatic interrogations of bacterial metabolism within the human microbiome.

The high prevalence of the *L. crispatus* PulA in CST I metagenomes (85%) further suggests an important role for this enzyme. Notably, in the *L. iners-*dominated CST III samples, the homologous PulA enzyme is much less prevalent (42%), perhaps suggesting that *L. iners* is less dependent on glycogen or relies more on other enzymes. Moreover, glycogen degradation has never been reported for *L. gasseri* or *L. jensennii* and we found no PulA homologues encoded in their genomes, leaving open questions about glycogen metabolism in CST II and V.

A major challenge in characterizing glycogen metabolism in clinical samples has stemmed from difficulties distinguishing human and bacterial amylase activity^21^. Our use of ABPP categorically identifies active enzymes in these complex samples. In addition, our simple LC–MS-based assay for pullulanase activity rapidly identifies bacterial GDE activity. Our results and those from other recent efforts^17,21^ show that bacterial GDE activity is highly variable, highlighting a need to test larger numbers of better-characterized clinical samples. We anticipate the pullulanase activity assay will find broad utility in the analysis of such samples and enable further study of the biological roles of bacterial GDEs^57^.

Overall, the insights gained from this investigation highlight the need to complement bioinformatic analysis with detailed biochemical characterization of vaginal bacterial enzymes. This improved understanding of the activities of vaginal bacterial GDEs will enable future exploration of bacterial glycogen metabolism in the vaginal microbiome and its contribution to community composition, stability and dysbiosis.

## Methods

### Institutional Review Board approval

This work complied with all relevant ethical regulations, and we obtained informed consent from all donors. The study protocols were approved by Massachusetts General Hospital (IRB: 2014P001066) and Seattle University Affiliates (IRB: FY2022‐002).

### Identification and cloning of glycogen-degrading enzymes

Homologues of PulA in *L. crispatus*^22^ (EEU28204.2) were identified by BLASTp searches of genomes from vaginal isolates in the IMG database^58^ using an *E*-value cut-off of 1 × 10^−5^. The IMG database contains 151 vaginal isolate genomes from the Human Microbiome Project with a sample body subsite of ‘vaginal’ (Supplementary File 1). Hits with no predicted signal peptide were removed (SignalP v.5.0 (ref. ^59^)). Six candidates with >35% amino acid identity from microbes associated with health or disease were selected. Genomic DNA was extracted from the encoding strains with a DNeasy UltraClean microbial kit (Qiagen). Genes were amplified via PCR removing the signal peptide (Supplementary Fig. 1) and cloned into the *E. coli* expression vector pET28a (Novagen) via Gibson assembly to generate an N-terminal His_6_-tagged gene. Plasmids were then transformed into the expression host BL21 (DE3) (*P. bivia* enzymes) or ArcticExpress (DE3) (all other enzymes) for expression and purification. Complete lists of plasmids and primers are provided in Supplementary Table 2 and Supplementary File 1, respectively.

### Purification of GDEs

Cultures containing expression plasmids were grown in LB medium containing 50 μg ml^−1^ kanamycin to an optical density at 600 nm (OD_600_) of 0.6–0.8, then cooled to 15 °C and induced with 250 μM isopropyl β-D-1 thiogalactopyranoside (IPTG). After 16 h at 15 °C, cells were collected and the pellets were stored at −20 °C until use. Pellets were resuspended in 98% buffer A (50 mM HEPES, 300 mM KCl, 10% glycerol, pH 7.8) and 2% buffer B (50 mM HEPES, 300 mM KCl, 10% glycerol, 500 mM imidazole, pH 7.8) supplemented with EDTA-free protease inhibitor cocktail (Sigma). Cells were lysed via homogenization (3 ×15,000 psi, Emulsiflex-C3, Avestin) and lysates were clarified (16,000 × *g* for 45 min at 4 °C) before being loaded onto a 5 ml HisTrap column (GE Healthcare). This was followed by one column volume (c.v.) of 2% buffer B and 2 c.v. of 10% buffer B. Protein was eluted using a linear gradient from 10 to 100% buffer B over 20 c.v. Protein-containing fractions and purity were determined by SDS–PAGE. Amylase-containing fractions were pooled, concentrated to a volume of ~1 ml in a spin concentrator (Millipore) and purified by size exclusion chromatography (GE Healthcare, Superdex 200) in 100% buffer A.

Fractions were again analysed by SDS–PAGE and protein-containing fractions were pooled, concentrated (Millipore, Amicon 30 kDa), flash frozen in liquid nitrogen and stored at −80 °C until use. Protein concentration was determined using a Bradford assay.

### *L. crispatus* growth recovery assay with purified protein

MRS broth containing glucose (BD Difco) was prepared according to manufacturer protocol. For growth assays on different carbon sources, MRS broth without glucose (Food Check Systems, pH 6.5–6.6) was prepared according to the manufacturer’s recipe and supplemented with either 2% d-glucose (Sigma), 2% maltose monohydrate (VWR) or 5% glycogen from oyster (Sigma). Each medium type was filter sterilized (0.2 μm) and left inside an anaerobic chamber with an atmosphere of 2.5% H_2_, 5% CO_2_ and 92.5% N_2_ (Coy Labs) overnight for equilibration. Starter cultures of *L. crispatus* C0176A1 and *L. crispatus* MV-1A-US were inoculated into MRS media (BD Difco) in Hungate tubes and incubated overnight at 37 °C. The next day, purified protein was thawed and added to 5% glycogen MRS media to a concentration ranging between 200–400 nM. The medium was again filter sterilized before use. As a negative control, protein boiled at 100 °C for 15 min was included. Of each medium type, 50 μl was aliquoted into a 384-well tissue culture-treated clear microplate (Corning). Overnight culture (1 μl) was used to inoculate each well. The plate was sealed and growth was monitored in a plate reader (Biotek) inside an anaerobic chamber (Coy Labs) at 37 °C for 24 h by measuring OD_600_ every 15 min.

### Kinetic analysis of GDEs

Kinetic analysis of GDEs was performed using a reducing sugar assay^41^, modified for a 96-well format. Reactions (300 μl) were set up containing substrate (0.0048–10 mg ml^−1^ glycogen; 0.0012–1.25 mg ml^−1^ Pullulan (Megazyme); or 0.0048–1.25 mg ml^−1^ amylose in a final concentration of 2% dimethyl sulfoxide), 0.8–700 nM enzyme and reaction buffer (20 mM sodium acetate, pH 5.5, 0.5 mM CaCl_2_). Reaction mixtures were incubated at 37 °C for 15 min and 50 μl aliquots were removed (2, 5, 7.5, 10, 15 min) into 125 μl of the BCA stop solution (0.4 M sodium carbonate, pH 10.7, 2.5 mM CuSO_4_, 2.5 mM 4,4’-dicarboxy-1,2’-biquinoline, 6 mM l-serine). After 30 min incubation at 80 °C, absorbances were read at 540 nm and compared to a maltose standard curve (0.000610–0.625 mg ml^−1^) to quantify activity. Initial velocities were calculated via linear regression, selecting the data points that produced the highest initial rate, utilizing at least three data points. *K*_M_ and *k*_cat_ parameters were determined by fitting the Michaelis–Menten equation using nonlinear regression (Graphpad Prism 8).

### Thin layer chromatography of enzymatic reactions with pullulan

A 1 μl volume from the GDE reaction mixtures were spotted onto a TLC plate and run for ~5 h in 3:2:1 butanol:acetic acid:water. The plate was removed, dried for 10 min with a heat gun and sprayed with a 1:19 sulfuric acid:ethanol solution. The plate was developed by heating for 15 min with a heat gun until spots appeared. Identity of products was confirmed by co-running pure standards.

### Enzyme pH profile and *G. vaginalis* PulA active site mutant activity on glycogen

Reactions were conducted using the reducing sugar assay (see above) with 1.25 mg ml^−1^ glycogen, 0.9–850 nM enzyme and assay buffer ranging in pH from 2.5 to 8.0 (pH 2.5–3.3: 20 mM glycine, 0.5 mM CaCl_2_; pH 4.0–5.5: 20 mM sodium acetate, 0.5 mM CaCl_2_; pH 6.0–6.5: 20 mM MES, 0.5 mM CaCl_2_; pH 7.0–8.0: 20 mM HEPES, 0.5 mM CaCl_2_). *G. vaginalis* PulA active site mutants were constructed using a multifragment Gibson assembly amplified from the wild-type expression vector pETpullGV using the primers listed in Supplementary File 1. pETpullGV-AS1 (∆AS1) and pETpullGV-AS2 (∆AS2) contained a D233A and D1317A mutation, respectively, designed to inactivate the catalytic aspartate of the amylase domains of these proteins. pETpullGV-DM (∆DBL) contained both mutations. Specific activities of *G. vaginalis* PulA active site mutants were determined at pH 5.5.

### Polysaccharide breakdown product analysis and growth studies

Reactions were set up containing 10 mg ml^−1^ substrate and 500 nM enzyme, all dissolved in reaction buffer and incubated at 37 °C overnight. Samples were quenched by 10-fold dilution into 90% acetonitrile. The plates were centrifuged (3,220 × *g* for 10 min, 4 °C) and the samples were diluted 1,000-fold in acetonitrile before analysis by UHPLC–MS using a Xevo TQ-S (Waters) with electrospray ionization (ESI). Sample (5 μl) was injected onto an Acquity BEH/Amide UPLC column (Waters, 1.7 µm, 130 Å, 2.1 mm × 50 mm) heated to 40 °C. A flow rate of 0.5 ml min^−1^ was used, with the following gradient: 0–1.0 min at 97% B (acetonitrile with 0.1% formic acid) and 3% A (H_2_O with 0.1% formic acid) isocratic, 1.0–4.0 min 97–30% B, 4.0–5.0 min at 30% B isocratic, 5.0–5.1 min at 30–97% B, 5.1–7.0 min at 97% B isocratic. Carbohydrate products were detected by ESI in positive mode (capillary voltage 3.10 kV; cone voltage 42 V; source offset voltage 50 V; desolvation temperature 500 °C; desolvation gas flow 1,000 l h^−1^; cone gas flow 150 l h^−1^; nebulizer 7.0 bar). See Supplementary Information for compound-specific detection parameters (Supplementary Table 3). For quantification of the oligosaccharides and their isomers, standards of glucose, maltose (VWR), maltotriose (Carbosynth), maltotetraose (Carbosynth) and maltopentaose (Carbosynth) were prepared ranging from 0.001–10 μg ml^−1^ in 9:1 acetonitrile:water. Oligosaccharide peak areas were quantified using the standard curve and the data were normalized to a no-enzyme control to account for non-enzymatic substrate breakdown (Waters MassLynx).

### Growth assays with amylase inhibitors

Growth inhibition assays were performed in an anaerobic chamber (Coy Labs) with an atmosphere of 2.5% H_2_, 5% CO_2_ and 92.5 N_2_. Bacteria were inoculated from single colonies into a peptone-yeast extract base broth (PYTs, pH 7.0–7.2) consisting of proteose peptone (20 g l^−1^), yeast extract (10 g l^−1^), MgSO_4_ (0.008 g l^−1^), K_2_HPO_4_ (0.04 g l^−1^), KH_2_PO_4_ (0.04 g l^−1^), NaHCO_3_ (0.4 g l^−1^), vitamin K (0.0025 g l^−1^), hemin (0.005 g l^-1^), l-cysteine • HCl (0.25 g l^−^^1^), Tween 80 (0.25 ml l^−1^), horse serum (50 ml l^−1^) and glucose (2 g l^−1^) and incubated at 37 °C for ~24 h. Cultures were adjusted to OD_600_ of 0.4–0.5, subcultured at a 1:50 dilution into fresh PYTs (without glucose), with the indicated carbohydrates added to a final concentration of 2 g l^−1^. Glycogen was from oyster (Sigma, G8751). Assays were performed in duplicate in 384-well plates sealed with BreathEasy gas permeable membranes (Diversified Biotech) under anaerobic conditions. Bacterial growth was monitored by measuring the OD_600_ at 1 h intervals for 48 h in a BioTek Epoch2 plate reader. Data were normalized to blank (uninoculated) media. For inhibition assays, bacteria were cultivated as above, with the addition of acarbose at the indicated concentrations. The extent of inhibition was determined by normalizing OD_600_ for each treatment to an untreated control at the time the control reached stationary phase, then IC_50_ values were calculated using least-squares regression (GraphPad Prism 8).

### Metagenomics and metatranscriptomics

ShortBRED was used to quantify the abundance of the six biochemically characterized bacterial GDEs in previously sequenced vaginal metagenomes and metatranscriptomes^49^. First, ShortBRED-Identify was used to create markers for all 6 PulA sequences using UniRef90 2017 as a reference list (Supplementary File 1) and an 85% cluster ID setting. Markers were used in ShortBRED-Quantify to determine the abundance of *pulA* genes and transcripts in paired metagenome and metatranscriptome databases (Bioproject PRJNA797778). The scripts used for processing the datasets have been previously described^48^. The output from ShortBRED-Quantify is reads per million reads per kilobase million (RPKM) and this was normalized to counts per microbial genome using the average genome sizes (AGS) of each metagenome sample, calculated using MicrobeCensus^60^. We normalized the output from ShortBRED using the previously derived equation shown below^61^.

$${\rm{Abundance}}={\rm{RPKM}}\times{\rm{AGS}}\times{10}^{-9}$$

Sample metadata were used to bin the results by community state type (CST I *n* = 39, CST II *n* = 16, CST III *n* = 31, CST IV *n* = 83, CST V *n* = 9). The fraction of samples positive for a bacterial GDE gene in a given CST was calculated by dividing the number of samples that contained a hit (reads >0) by the total number of samples with the corresponding CST.

### CVL analysis for amylase activity and human amylase abundance

CVLs were obtained from Dr Caroline Mitchell at Massachusetts General Hospital (IRB: 2014P001066). All metadata associated with this cohort are reported (Supplementary File 1). CVLs were collected using 3 ml of sterile saline washed over the cervix and vaginal walls with a transfer pipette and then re-aspirated. Samples were centrifuged (10,000 × *g* for 10 min at 4 °C) and the supernatants were decanted and used in the assay. Purified proteins were diluted in buffer A (50 mM HEPES, 300 mM KCl, 10% glycerol, pH 7.8) to 1 μM, then used in the assay. Human salivary amylase was purchased from Sigma Aldrich (A1031-1KU). Human amylase was detected in CVLs using an ELISA for human pancreatic amylase (Abcam ab137969) according to manufacturer instructions.

Amylase activity of CVL supernatants was determined using the EnzCheck Ultra Amylase Assay kit (Thermo Fisher, E33651). The substrate was prepared according to the kit instructions using three different buffers (20 mM sodium acetate, 0.5 mM CaCl_2_, pH 4.4; 20 mM sodium acetate, 0.5 mM CaCl_2_, pH 5.5; 20 mM MES, 0.5 mM CaCl_2_, pH 6.8). CVL supernatant (10 μl) was added to each well of a black clear-bottom 96-well plate and then diluted with 40 μl of pH-adjusted buffer. The reactions were initiated with 50 μl of substrate and incubated for 30 min at 37 °C. The pH-adjusted buffer made up 90% of the reaction volume and each kit reagent was dissolved in the corresponding buffer. Fluorescence was measured at 485/528 nm. Initial rates were calculated in the plate reader software (Biotek) by determining the highest slope that covered at least 5 data points.

### Activity-based protein profiling in CVF samples

CVF samples were collected from Seattle University Affiliates (IRB: FY2022‐002). The participants were not compensated for their inclusion in the study. All metadata are reported in Supplementary Information (Supplementary Table 4). Donors self-collected a sample by inserting a Soft Disc and then waiting 1–4 h before removing the disc and placing it into a 50 ml conical vial. Within 1 h of collection, CVF was removed from the disc through the addition of 1 ml PBS and centrifugation at 200 × *g* for 8 min. Samples were then frozen in 0.1 ml aliquots at −70 °C.

Biotinylated and fluorescent probes for α-amylases (CYR1114 and CYR232, respectively)^26^ and α-glucosidases (JJB384 and JJB383, respectively)^62^ were kindly provided by Dr Hermann Overkleeft and Dr Gideon Davies (Leiden University). Before use, CVF samples were spun down (10,000 × *g* for 5 min) to remove mucins. CVF samples were normalized to a protein concentration (bicinchoninic acid assay) of 1 mg ml^−1^ using sterile PBS, and EDTA-free protease inhibitor cocktail (Roche) was added. CVF supernatant was then incubated with the fluorescent amylase probe at a final concentration of 25 μM or the fluorescent glucosidase probe at a final concentration of 10 μM. Negative controls of vehicle (1% v/v dimethyl sulfoxide in water) and heat-shock controls were included to identify background fluorescence or off-target labelling. Samples were incubated for 4 h at 37 °C. Proteins were then separated on a 4–20% PAGE gel (Bio-Rad) and probe fluorescence was visualized (Azure C600).

Active amylases were enriched using ABPP and identified via LC–MS/MS as previously described, with slight modifications^50^. CVF supernatant prepared as above was divided into three 400 μl aliquots. Biotinylated amylase probe (final concentration 25 μM), biotinylated glucosidase probe (final concentration 10 μM) or an equal volume of vehicle (1% dimethyl sulfoxide in water) was added and samples were incubated for 4 h at 37 °C. After labelling, 400 μl of ice-cold methanol was added and samples were stored at −70 °C overnight to precipitate proteins. Precipitated protein was collected via centrifugation (10,000 × *g* for 10 min), redissolved in 500 μl 1.2% SDS in PBS and heated at 95 °C for 2 min. Samples were then centrifuged (14,000 × *g* for 5 min) to remove insoluble proteins.

Streptavidin agarose resin (100 μl, Thermo Fisher) was prepared by washing with 0.5% w/v SDS in PBS (3×), 6 M urea in 25 mM NH_4_HCO_3_ (3×) and PBS (3×) using a vacuum manifold. Washed resin in 2 ml of PBS was then added to protein samples and samples were incubated, rotating at 37 °C for 1 h. Samples were then transferred to columns (Bio-Rad Poly-Prep) on a vacuum manifold and washed with 1 ml volumes of 0.5% w/v SDS in PBS (3×), 6 M urea in 25 mM NH_4_HCO_3_ (3×), ultrapure water (3×), PBS (9×) and 25 mM NH_4_HCO_3_ (5×). Resin was then transferred in 6 M urea in 25 mM NH_4_HCO_3_ to low-bind Eppendorf tubes and reduced with 5 mM DTT at 37 °C for 30 min, followed by alkylation with 10 mM iodoacetamide at 50 °C for 1 h. Samples were washed with PBS (9×) and 25 mM NH_4_HCO_3_ (5×). Resin was then transferred to new low-bind Eppendorf tubes, resuspended in 200 μl 25 mM NH_4_HCO_3_ and 0.4 μl 0.25 μg μl^−1^ trypsin (Promega, proteomics grade) in 25 mM HEPES was added. Samples were incubated overnight at 37 °C with rotation. Supernatants were collected followed by an additional resin wash with 150 μl 25 mM NH_4_HCO_3_, which was added to the original supernatant. The peptides were then dried down (Speed-vac) before further analysis.

Except for S010, LC–MS/MS analysis was performed with a Thermo Scientific Easy1200 nLC (Thermo Scientific) coupled to a tribrid Orbitrap Eclipse (Thermo Scientific) mass spectrometer. In-line desalting was accomplished using a reversed-phase trap column (100 μm × 20 mm) packed with Magic C18AQ (5 μm 200 Å resin; Michrom Bioresources), followed by peptide separations on a reversed-phase column (75 μm × 270 mm) packed with ReproSil-Pur C18AQ (3 μm 120 Å resin; Dr Maisch) directly mounted on the electrospray ion source. A 60 min gradient using a two-mobile-phase system consisted of 0.1% formic acid in water (A) and 80% acetonitrile in 0.1% formic acid in water (B). The chromatographic separation was achieved over a 60 min gradient from 8 to 30% B over 57 min, 30 to 45% B for 10 min, 45 to 60% B for 3 min, 60 to 95% B for 2 min and held at 95% B for 11 min at a flow rate of 300 nl min^−1^. A spray voltage of 2,300 V was applied to the electrospray tip in line with a FAIMS source using varied compensation voltages of –40, –60 and –80 while the Orbitrap Eclipse instrument was operated in the data-dependent mode, MS survey scans were in the Orbitrap (normalized AGC target value 300%, resolution 240,000 and maximum injection time 50 ms) with a 1 s cycle time, and MS/MS spectra acquisition were detected in the linear ion trap (normalized AGC target value of 50% and injection time 35 ms) using HCD activation with a normalized collision energy of 27%. Selected ions were dynamically excluded for 60 s after a repeat count of 1. For S010, peptide samples were disolved in 2% acetonitrile in 0.1% formic acid (20 μl) and analysed (18 μl) by LC/ESI MS/MS with a Thermo Scientific Easy-nLC 1000 (Thermo Scientific) coupled to a tribrid Orbitrap Fusion (Thermo Scientific) mass spectrometer. In-line desalting was accomplished using a reversed-phase trap column (100 μm × 20 mm) packed with Magic C_18_AQ (5 μm 200 Å resin; Michrom Bioresources), followed by peptide separations on a reversed-phase column (75 μm × 250 mm) packed with ReproSil-Pur 120 C_18_AQ (3 μm 120 Å resin Dr Maisch) directly mounted on the electrospray ion source. A 90-min gradient from 2% to 35% acetonitrile in 0.1% formic acid at a flow rate of 300 nl min^−1^ was used for chromatographic separations. A spray voltage of 2,200 V was applied to the electrospray tip and the Orbitrap Fusion instrument was operated in the data-dependent mode, MS survey scans were in the Orbitrap (AGC target value 500,000, resolution 120,000 and injection time 50 ms) with a 3 s cycle time and MS/MS spectra acquisition were detected in the linear ion trap (AGC target value of 10,000 and injection time 35 ms) using HCD activation with a normalized collision energy of 27%. Selected ions were dynamically excluded for 20 s after a repeat count of 1.

Samples were analysed with FragPipe IonQuant enabled^63–66^. Spectra were matched to a database containing UniProt human reference proteins; UniRef90 proteins for *L. crispatus*, *L. iners*, *L. gasseri*, *L. jensenii*, *G. vaginalis*, *A. vaginae*, *P. bivia* and *M. mueleris*; common contaminants; and reverse protein sequences as decoys for false discovery rate (FDR) estimation (accessed 25 May 2022). Raw data are available in Supplementary File 1. Abundance data were analysed using Perseus^67^. Abundance data were log_2_ transformed and normalized using width adjustment. For S010, protein groups present in two of three replicates were averaged and the data tables were combined. Proteins with at least a 2-fold increased abundance relative to the No Probe control in one biological sample, 2 spectral counts across all samples and a ProteinProphet probability >0.95 (corresponding to ~2% FDR) were searched for CAZyme domains using dbCAN 2 (ref. ^68^).

### Pullulanase activity assays in CVF samples

CVF fluid (5 μl, not centrifuged) was added to 95 μl 10 mg ml^−1^ pullulan (Megazyme) in reaction buffer. The reaction mixtures were incubated at 37 °C and timepoints at 3, 5, 8 and 24 h were taken by diluting 100-fold into 9:1 acetonitrile:water. Samples were further diluted 1,000-fold in acetonitrile and analysed by LC–MS as described above. Samples were normalized to a no-enzyme control.

### Inhibitor screening and IC_50_ determination for acarbose

The inhibitory effect of a panel of four small-molecule inhibitors was determined using a modification of the amylase activity assay in CVLs used above. For initial screening, enzymes were preincubated with 1 mM acarbose (Abcam), acarviosin (Toronto Research Products), voglibose (Spectrum Chemical) or miglitol (Tokyo Chemical) for 15 min at room temperature. For IC_50_ analysis, enzyme (2.5–50 nM) was preincubated with acarbose ranging from 0.366 μM to 3,000 μM in a total volume of 50 μl. The reactions were initiated with 50 μl of substrate and incubated for 30 min at 37 °C, monitoring fluorescence at 485/528 nm. Initial rates were calculated by determining the highest slope that covered at least 8 data points. Percent activity was calculated by normalizing the activity to a no-inhibitor control. IC_50_ values were calculated using nonlinear fitting of the data to the inhibitor vs normalized response function (GraphPad Prism 8). Error associated with the IC_50_ values represents 95% confidence intervals.

### Statistics and reproducibility

No statistical method was used to predetermine sample size for any of the statistical comparisons. However, our sample size was similar to previous work on this topic^21^. For all statistical tests, data distribution was assumed to be normal, but this was not formally tested. Randomization was not relevant to this study because we did not place participants into groups. A ROUTE test was used to identify and remove outliers in the activity analysis of the CVL samples (Extended Data Fig. 8). The researchers performing the activity analysis of the clinical samples were blinded to the metadata during the course of the study.

### Reporting summary

Further information on research design is available in the Nature Portfolio Reporting Summary linked to this article.

## Supplementary information

Supplementary Information Supplementary Figs. 1–5 and Tables 1–4.

Reporting Summary

Supplementary File 1

## Data Availability

The protein identification number in the NCBI database for each enzyme characterized is as follows: *L. crispatus* PulA (EEU28204.2), *L. iners* PulA (EFQ51965.1), *G. vaginalis* PulA (EPI56559.1), *M. mulieris* PulA (EEZ90738.1), *P. bivia* PulA (WP_061450340.1), *P. bivia* GH13 (WP_036862728.1). The *L. crispatus* C0176A1 (PulA^−^) genome can be found under accession number JAEDCG000000000. The metagenomic and metatranscriptomic datasets used in this study can be found under Bioproject PRJNA797778. The proteomics data from this study can be accessed in the PRIDE database using accession code PXD042917. Protein domain annotations were from the Pfam and CAZy databases. All data that support the findings of this study are available in a data repository at synapse.org and can be accessed at https://www.synapse.org/#!Synapse:syn51422003. Source data are provided with this paper.

## Source data

Source Data Fig. 1 Statistical source data.
Source Data Fig. 2 Statistical source data.
Source Data Fig. 3 Statistical source data.
Source Data Fig. 4 Statistical source data.
Source Data Table 1 Statistical source data.
Source Data Extended Data Fig. 1 Unprocessed SDS–PAGE gel.
Source Data Extended Data Fig. 2 Statistical source data.
Source Data Extended Data Fig. 3 Statistical source data.
Source Data Extended Data Fig. 4 Statistical source data.
Source Data Extended Data Fig. 4 Unprocessed SDS–PAGE gel.
Source Data Extended Data Fig. 6 Statistical source data.
Source Data Extended Data Fig. 7 Statistical source data.
Source Data Extended Data Fig. 8 Statistical source data.
Source Data Extended Data Fig. 10 Statistical source data.
