# Supplementary material for: Bacterial amylases enable glycogen degradation by the vaginal microbiome
Source: Nat Microbiol. 2023 Aug 10;8(9):1641–52. doi: 10.1038/s41564-023-01447-2 (PMC10465358; doi:10.1038/s41564-023-01447-2)
Supplement: Supplementary file 1 — Supplementary Figs. 1–5 and Tables 1–4. [file 41564_2023_1447_MOESM1_ESM.pdf]

nature microbiology

Article

https://doi.org/10.1038/s41564-023-01447-2

Bacterial amylases enable glycogen degradation by the vaginal microbiome

In the format provided by the authors and unedited

Supplementary information table of contents

Supplementary Figure 1. **Page 2**

Signal peptide removal for heterologous expression in E. coli.

Supplementary Figure 2. **Page 3**

CVL samples contain human amylase.

Supplementary Figure 3. **Page 4**

Human amylase presence and amylase activity do not correlate with Nugent score.

Supplementary Figure 4. **Page 5**

Chemical structures of the probes used for activity-based protein profiling.

Supplementary Figure 5. **Page 6**

Purified GDEs from vaginal bacteria react with ABPP probes.

Supplementary Table 1. **Page 7**

Strains used in this study.

Supplementary Table 2. **Page 8**

Plasmids used in this study.

Supplementary Table 3. **Page 9**

LC-MS compound specific detection parameters

Supplementary Table 4. **Page 10**

Metadata describing CVF sample donors used in ABPP and pullulanase assays.

```
                              10        20        30        40        50
L. crispatus PulA    MNKKSGHNIKFKSIFVCTSAIMSLWLGANLTTTQVHAAEDNAAPKSSEVV
L. iners PulA        MNFKINKKKITFLCTSTILGLVVSVSTVNADAVNNNIKNNNNVSLIKAPT
G. vaginalis PulA    MQMVAHRHLASKIVSMIAASAMLITGFAVVGNASASSESSSESSNPQSSN
M. mulieris PulA     MSVQRTKGLFSVLVAFFLVLAMQSLTAVNVAHADVGNMYLSFTPDPAIQK
P. bivia PulA        MRIRHYLVAAFASILLAQGVKAQDIFNEVSYTKQATTFCLNAPSKPVLRL
P. bivia GH13        MKKEAFLMILASLMFLTTNTKAQKRTTIDRIEPTNWYVGMQDPSLQLMIY
```

Supplementary Figure 1. Signal peptide removal for heterologous expression in *E. coli.* Blue text denotes the amino acid residues removed from each protein sequence during the cloning process.

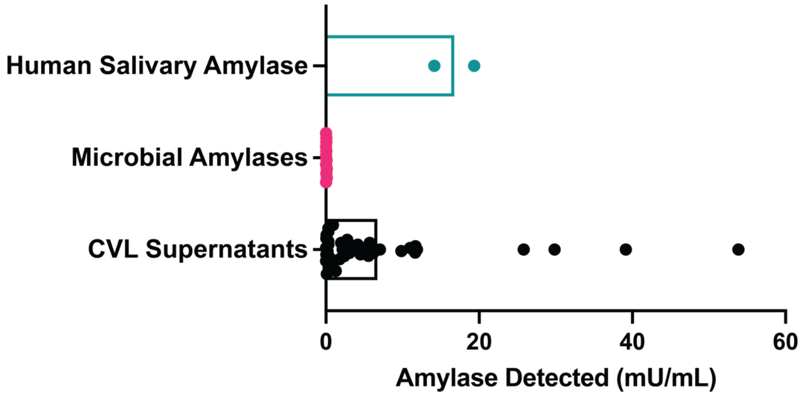

Supplementary Figure 2. CVL samples contain human amylase. ELISA detection for human pancreatic amylase in patient CVL supernatants, purified microbial GDEs (1 μM), and purified human salivary amylase (1 μM). 20 CVLs, six different microbial GDEs, and one human salivary amylase were measured in two experimental replicates over two days. All samples and replicates were plotted, and the mean of the samples is denoted by the bar.

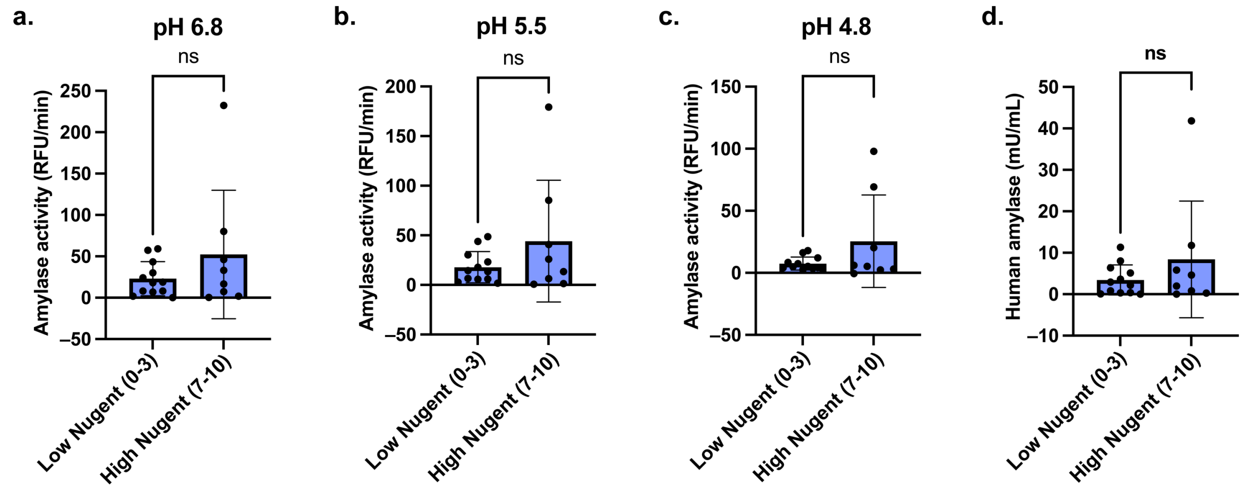

Supplementary Figure 3. Human amylase presence and amylase activity do not correlate with Nugent score. **a.** CVL activity levels measured using a fluorescent starch substrate at pH 6.8 stratified by Nugent score determination (P = 0.2249) **b.** CVL activity levels measured using a fluorescent starch substrate at pH 5.5 stratified by Nugent score determination (P = 0.1702) **c.** CVL activity levels measured using on a fluorescent starch substrate at pH 4.8 stratified by Nugent score determination (P = 0.1105) **d.** ELISA amylase detection of human amylase in CVL samples stratified by Nugent score determination (P = 0.2535). The X-axes of all plots are Nugent scores (Low Nugent (0-3), n=12; High Nugent (7-10), n=8). Error bars are representative of one standard deviation above and below the mean. A two-tailed non-parametric t-test was used to determine statistical significance. P-value symbols: P>0.05 (ns), P≤0.05 (*), P≤0.01 (**), P≤0.001 (***), P≤0.0001(****). All plots were created, and statistics performed in GraphPad Prism 8.

a.

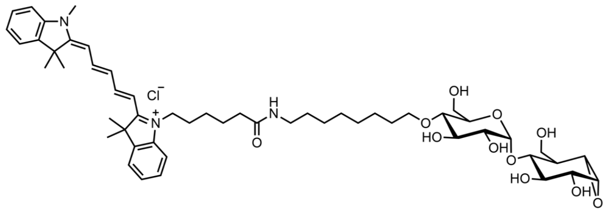

CYR232

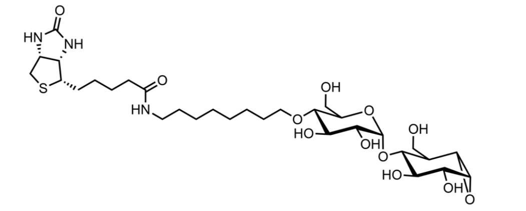

CYR1114

b.

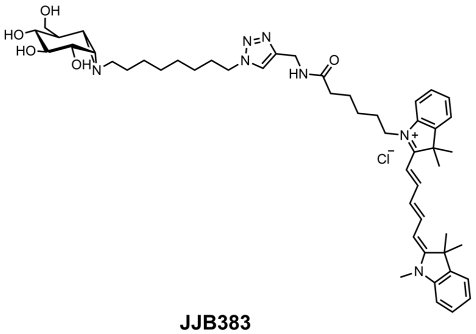

JJB383

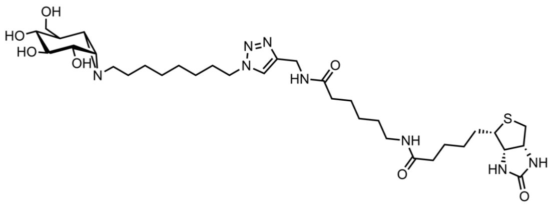

JJB384

Supplementary Figure 4. Chemical structures of the probes used for activity-based protein profiling. **a.** α-amylase probes (Amy-ABP) with Cy5 (left) and biotin (right) handles. **b.** α-glucosidase probes (Glc-ABP) with Cy5 (left) and biotin (right) handles.

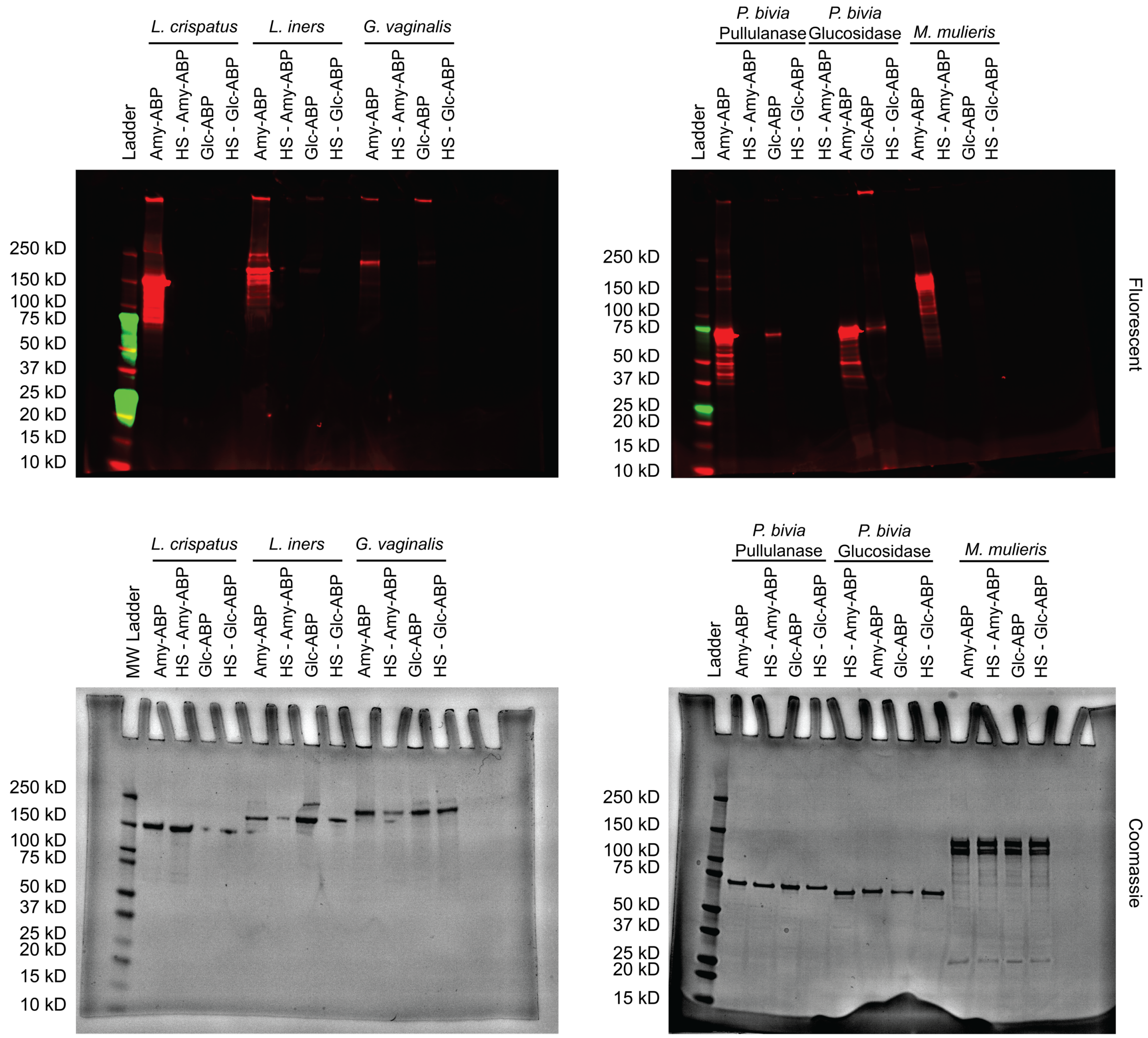

Supplementary Figure 5. Purified GDEs from vaginal bacteria react with ABPP probes. Purified GDEs were probed using fluorescent Amy-ABP, Glc-ABP, or vehicle only (**NP**, no probe; **HS**, heat shock) for 2 hours at 37° C and visualized on a 4-20% SDS-PAGE gel (Bio-Rad). Left lane: Precision Plus Protein WesternC standard (Bio-Rad). Fluorescence gel images (top) and Coomassie-stained image of the same gels (bottom) are representative of three independent experiments over three days.

Supplementary Table 1: Strains used in this study.

Name	Description / Use	Reference / Source
E. coli DH5α	Cloning strain	NEB (New England Biolabs)
E. coli BL21(DE3)	Protein expression and purification	NEB
E. coli Arctic Express (DE3)	Protein expression with co-expression of psycrophilic chaperones	Agilent
Lactobacillus crispatus MV-1A-US	Source of *pulA* gene, Growth Studies	BEI
Lactobacillus iners LEAF 3008A-a	Source of *pulA* gene, Growth Studies	BEI
Gardnerella vaginalis JCP7276	Source of *pulA* gene, Growth Studies	BEI
Mobiluncus mulieris str. 28-1	Source of *pulA* gene, Growth Studies	BEI
Prevotella bivia GED7760C	Source of *pulA* gene, Growth Studies	BEI
Lactobacillus crispatus C0176A1	Growth Studies	VMRC

Supplementary Table 2: Plasmids used in this study.

Name	Description / Use	Reference / Source
pET28a	ColE1 lacI pT7 Kn^R. Expression vector	Novagen
pETpullLC	pET28 with N-HIS-tagged *L. crispatus pulA*	This work
pETpullGV	pET28 with N-HIS-tagged *G. vaginalis pulA*	This work
pETpullLI	pET28 with N-HIS-tagged *L. iners pulA*	This work
pETpullMM	pET28 with N-HIS-tagged *M. mulieris pulA*	This work
pETpullPB	pET28 with N-HIS-tagged *P. bivia pulA*	This work
pETgh13PB	pET28 with N-HIS-tagged *P. bivia glycosidase*	This work
pETpullGV-AS1	pETpullGV with D233A mutation (based on numbering of native protein sequence)	This work
pETpullGV-AS2	pETpullGV with D1317A mutation	This work
pETpullGV-DM	pETpullGV with D233A and D1317A mutations	This work

Supplementary Table 3: LC-MS compound specific detection parameters.

**Compound**	**Mass *(m/z)***	**Cone Voltage (volts)**	**Elution time (min)**
Glucose	203.0	47	3.53
Maltose	365.0	76	3.85
Maltotriose	527.1	98	4.03
Maltotetraose	689.1	100	4.15
Maltopentaose	851.3	100	4.28

Supplementary Table 4: Metadata describing CVF sample donors used in ABPP and pullulanase assays.

**Sample**	**Age Range**	**Race/Ethnicity**	**Hormonal Contraceptive Use**	**Day of Cycle**
S003	18-25	Hispanic/White	No	9
S004	18-25	White	No	16
S006	18-25	White	No	21
S008	18-25	White	Yes	15
S010	18-25	Asian/White	Yes	NA
S011	18-25	Hispanic	Yes	NA
