# Supplementary material for: Bacterial amylases enable glycogen degradation by the vaginal microbiome
Source: Nat Microbiol. 2023 Aug 10;8(9):1641–52. doi: 10.1038/s41564-023-01447-2 (PMC10465358; doi:10.1038/s41564-023-01447-2)
Supplement: Supplementary file 2 — Reporting Summary [file 41564_2023_1447_MOESM2_ESM.pdf]

nature portfolio

Corresponding author(s): Seth Rakoff Nahoum
Christopher Whidbey
Emily P. Balskus

Last updated by author(s): Jun 30, 2023

Reporting Summary

Nature Portfolio wishes to improve the reproducibility of the work that we publish. This form provides structure for consistency and transparency in reporting. For further information on Nature Portfolio policies, see our Editorial Policies and the Editorial Policy Checklist.

Statistics

For all statistical analyses, confirm that the following items are present in the figure legend, table legend, main text, or Methods section.

n/a	Confirmed	
☐	☒	The exact sample size (n) for each experimental group/condition, given as a discrete number and unit of measurement
☐	☒	A statement on whether measurements were taken from distinct samples or whether the same sample was measured repeatedly
☐	☒	The statistical test(s) used AND whether they are one- or two-sided<br>*Only common tests should be described solely by name; describe more complex techniques in the Methods section.*
☒	☐	A description of all covariates tested
☐	☒	A description of any assumptions or corrections, such as tests of normality and adjustment for multiple comparisons
☐	☒	A full description of the statistical parameters including central tendency (e.g. means) or other basic estimates (e.g. regression coefficient) AND variation (e.g. standard deviation) or associated estimates of uncertainty (e.g. confidence intervals)
☐	☒	For null hypothesis testing, the test statistic (e.g. F, t, r) with confidence intervals, effect sizes, degrees of freedom and P value noted<br>*Give P values as exact values whenever suitable.*
☒	☐	For Bayesian analysis, information on the choice of priors and Markov chain Monte Carlo settings
☒	☐	For hierarchical and complex designs, identification of the appropriate level for tests and full reporting of outcomes
☐	☒	Estimates of effect sizes (e.g. Cohen's d, Pearson's r), indicating how they were calculated

Our web collection on statistics for biologists contains articles on many of the points above.

Software and code

Policy information about availability of computer code

Data collection	The publicly available IMG BLAST tool (NCBI+ 2.10) was used for informatics searches for pulA homologs. Mass spec data was collected using Waters MassLynx (v4.2). Proteomics data was collected on an Thermo Scientific Easy1200 nLC (Thermo Scientific) coupled to a tribrid Orbitrap Eclipse (Thermo Scientific) mass spectrometer and an by LC/ESI MS/MS with a Thermo Scientific Easy-nLC 1000 (Thermo Scientific) coupled to a tribrid Orbitrap Fusion (Thermo Scientific) mass spectrometer. Shortbred was used to identify microbial enzymes in metagenomic and metatranscriptomic sequencing datasets (Shortbred 0.9.5).
Data analysis	Graphpad Prism (v8.2.1) was used for kinetic analysis and regression. FragPipe IonQuant (v1.8.0) was used for proteomics analysis

For manuscripts utilizing custom algorithms or software that are central to the research but not yet described in published literature, software must be made available to editors and reviewers. We strongly encourage code deposition in a community repository (e.g. GitHub). See the Nature Portfolio guidelines for submitting code & software for further information.

Data

Policy information about availability of data

All manuscripts must include a data availability statement. This statement should provide the following information, where applicable:

- Accession codes, unique identifiers, or web links for publicly available datasets
- A description of any restrictions on data availability
- For clinical datasets or third party data, please ensure that the statement adheres to our policy

The protein identification number in the NCBI database for each enzyme characterized is as followed, L. crispatus PulA (EEU28204.2), L. iners PulA (EFQ51965.1), G. vaginalis PulA (EPI56559.1), M. mulieris PulA (EEZ90738.1), P. bivia PulA (WP_061450340.1), P. bivia GH 13 (WP_036862728.1). The L. crispatus C0176A1 (PulA-) genome can be found under the following accession number JAEDCG000000000. The metagenomic and metatranscriptomic datasets used in this study can be found under the Bioproject PRJNA797778. The proteomics data from this study can be accessed in the PRIDE database using the accession code PXD042917. Protein domain annotations were from the Pfam and CAZy databases. All data that supports the findings of this study will be available in a data repository at synapse.org. It can be accessed using the following link https://www.synapse.org/#!Synapse:syn51422003.

Human research participants

Policy information about studies involving human research participants and Sex and Gender in Research.

Reporting on sex and gender

This cohort was enrolled to study the vaginal microbiome and mucosal immunology, thus only people with a vagina were enrolled.

Population characteristics

Population characteristics for each cohort can be found in supplementary table 4 and 5.

Recruitment

For the study conducted at Massachusetts General Hospital (IRB: 2014P001066), participants were recruited in the following ways:

a. Informational flyers were placed in the gynecology clinics with information about the study, to introduce the idea to patients. Contact information was given for the principal investigator so that patients can ask questions about the study.

b. Letters were sent to the gynecology staff to describe the study, and contact information provided for the principal investigator so that staff can ask questions about the study.

c. Patients presenting to gynecology clinic either for vulvovaginitis specialty care, or for an annual exam, were offered informational flyers about the study and approached by the research coordinator while in a room waiting to be seen to ask if they are interested in hearing more about the study.

People who volunteer for research studies focused on vaginal microbiome may be more likely to have symptoms and/or abnormal microbial communities. The diagnosis for every participant is listed in Supplementary File 1. Both studies recruited people within specific institutions, which may limit the population of people who learn about the study and have the opportunity to participate.

For the study conducted at Seattle University (IRB: FY2022-002), participants were recruited from Seattle University affiliates via email, social media, and announcements in classes. Potential donors were asked to contact the research team via email.

For ABPP experiments all volunteers were undergraduates at Seattle University between 18-25 years old. Given that the vaginal physiology changes with age, these findings can't necessarily be extrapolated to pre-menarche or post-menopause individuals. Because of the small sample size, it is difficult to draw conclusions regarding enzyme activity profile and race/ethnicity. Demographic data regarding race/ethnicity are provided in Supplementary Table 4.

Ethics oversight

Massachusetts General Hospital (IRB: 2014P001066) and Seattle University (IRB: FY2022-002)

Note that full information on the approval of the study protocol must also be provided in the manuscript.

Field-specific reporting

Please select the one below that is the best fit for your research. If you are not sure, read the appropriate sections before making your selection.

☒ Life sciences ☐ Behavioural & social sciences ☐ Ecological, evolutionary & environmental sciences

For a reference copy of the document with all sections, see nature.com/documents/nr-reporting-summary-flat.pdf

Life sciences study design

All studies must disclose on these points even when the disclosure is negative.

Sample size

No statistical method was used to predetermine sample size for any of the statistical comparison. The sample size was based on how many

Sample size	samples were available in the cohorts that were recruited. However our sample size is similar to other clinical cohorts studying this topic (https://doi.org/10.1128/msphere.00943-20 (N=23), https://doi.org/10.1101/2022.03.29.486257 (N=17)).
Data exclusions	A ROUTE test was applied in Extended data Fig. 8 based on a reviewer's request to remove outliers that were dominating the regression analysis.
Replication	Each of the experiments was repeated at least three different times for statistical comparisons unless noted in the manuscript. All attempts at replication were successful.
Randomization	Randomization was not relevant to this study because we did not place participants into groups.
Blinding	Blinding was not relevant in this study, because methods used in the assessment of the results were objective.

Reporting for specific materials, systems and methods

We require information from authors about some types of materials, experimental systems and methods used in many studies. Here, indicate whether each material, system or method listed is relevant to your study. If you are not sure if a list item applies to your research, read the appropriate section before selecting a response.

Materials & experimental systems

n/a	Involved in the study	
☐	☒	Antibodies
☒	☐	Eukaryotic cell lines
☒	☐	Palaeontology and archaeology
☒	☐	Animals and other organisms
☒	☐	Clinical data
☒	☐	Dual use research of concern

Methods

n/a	Involved in the study	
☒	☐	ChIP-seq
☒	☐	Flow cytometry
☒	☐	MRI-based neuroimaging

Antibodies

Antibodies used	The antibiody used was part of a commercial ELISA kit from Abcam (ab137969)
Validation	Abcam ELISA antibody performance is validated by the manufacturer through spike-recovery experiments in a variety of biological matrices, and linearity studies, as described at https://www.abcam.com/primary-antibodies/how-we-validate-our-antibodies#ELISA
