# Supplementary figures and images for: Bacterial amylases enable glycogen degradation by the vaginal microbiome
Source: Nat Microbiol. 2023 Aug 10;8(9):1641–52. doi: 10.1038/s41564-023-01447-2 (PMC10465358; doi:10.1038/s41564-023-01447-2)

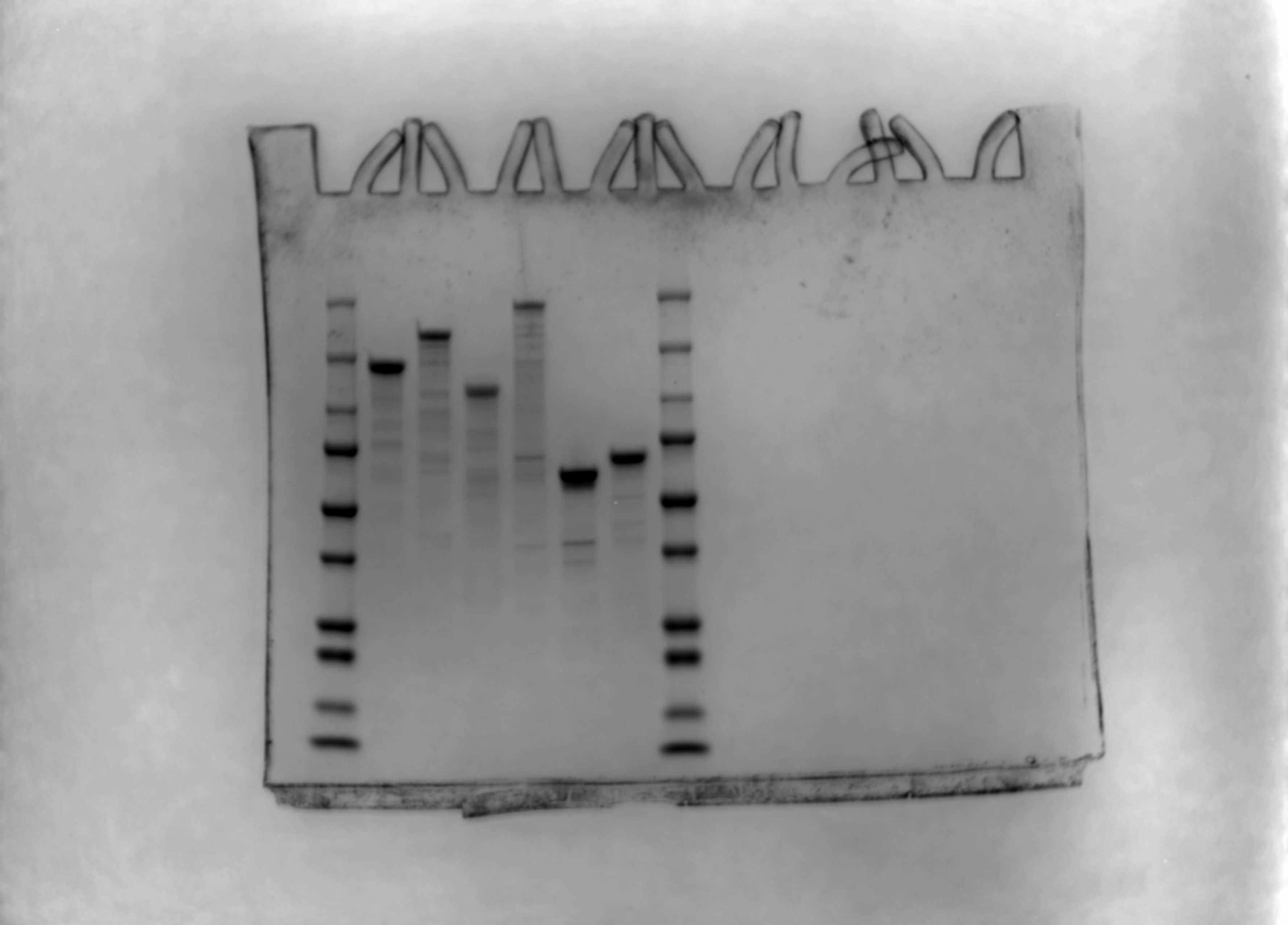

Supplement: Source Data Extended Data Fig. 1 — Unprocessed SDS–PAGE gel. [file 41564_2023_1447_MOESM9_ESM.jpg]

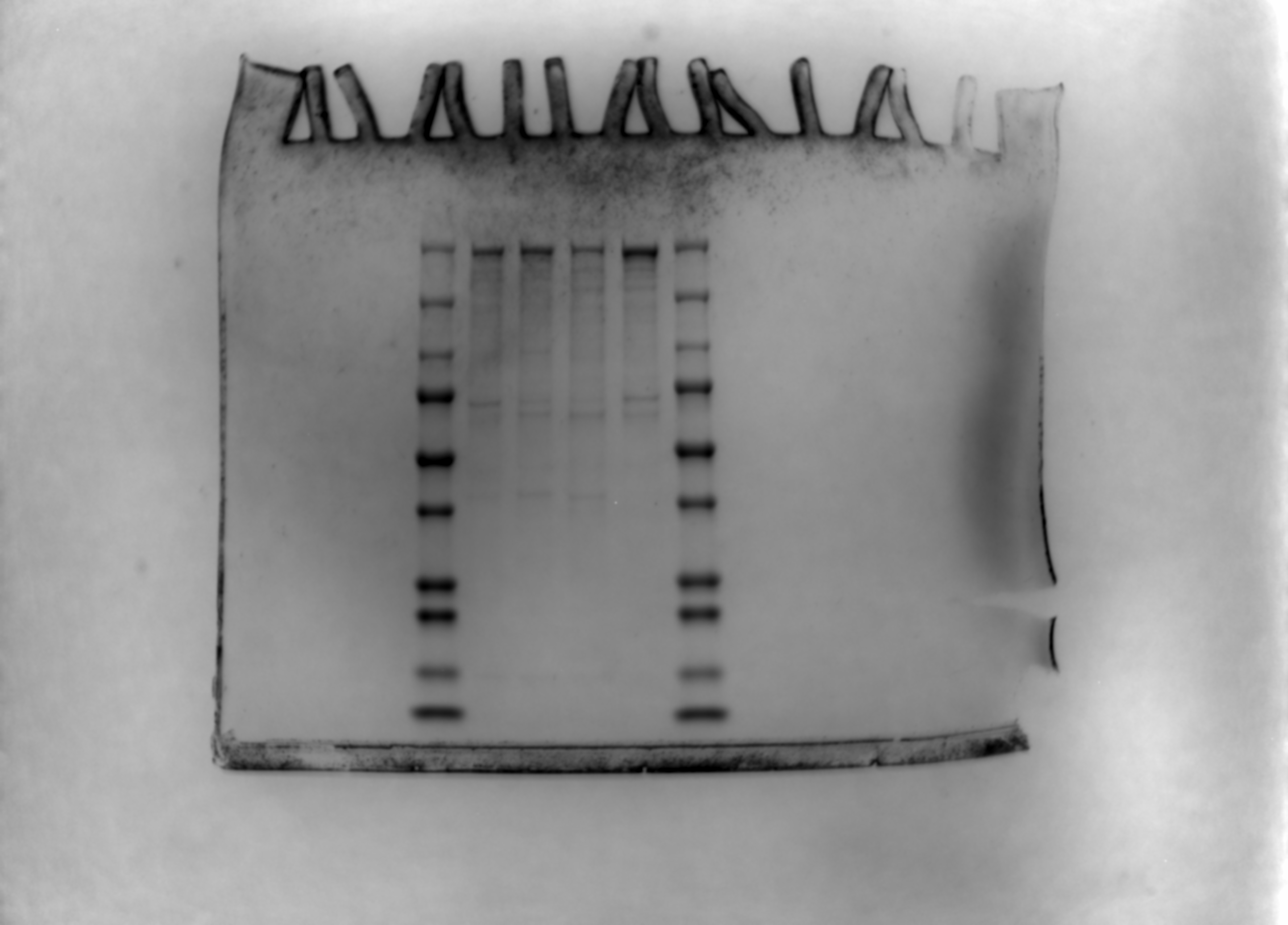

Supplement: Source Data Extended Data Fig. 4 — Unprocessed SDS–PAGE gel. [file 41564_2023_1447_MOESM13_ESM.jpg]
